# A reference genome for *Nicotiana tabacum* enables map-based cloning of homeologous loci implicated in nitrogen utilization efficiency

**DOI:** 10.1186/s12864-017-3791-6

**Published:** 2017-06-19

**Authors:** K. D. Edwards, N. Fernandez-Pozo, K. Drake-Stowe, M. Humphry, A. D. Evans, A. Bombarely, F. Allen, R. Hurst, B. White, S. P. Kernodle, J. R. Bromley, J. P. Sanchez-Tamburrino, R. S. Lewis, L. A. Mueller

**Affiliations:** 1 Plant Biotechnology Division, British American Tobacco, Cambridge, UK; 2 Boyce Thompson Institute, Ithaca, NY USA; 3 Crop Science Department, North Carolina State University, Raleigh, NC USA; 4 Present address Department of Horticulture, Virginia Tech, Blacksburg, VA USA

**Keywords:** Sequencing, *Nicotiana*, *Nicotiana tabacum*, tobacco, *Solanaceae*, Nitrogen use efficiency, Nitrogen utilization efficiency, *EGY1*, Map-based cloning, Polyploidy

## Abstract

**Background:**

Tobacco (*Nicotiana tabacum*) is an important plant model system that has played a key role in the early development of molecular plant biology. The tobacco genome is large and its characterisation challenging because it is an allotetraploid, likely arising from hybridisation between diploid *N. sylvestris* and *N. tomentosiformis* ancestors. A draft assembly was recently published for *N. tabacum*, but because of the aforementioned genome complexities it was of limited utility due to a high level of fragmentation.

**Results:**

Here we report an improved tobacco genome assembly, which, aided by the application of optical mapping, achieves an N_50_ size of 2.17 Mb and enables anchoring of 64% of the genome to pseudomolecules; a significant increase from the previous value of 19%. We use this assembly to identify two homeologous genes that explain the differentiation of the burley tobacco market class, with potential for greater understanding of Nitrogen Utilization Efficiency and Nitrogen Use Efficiency in plants; an important trait for future sustainability of agricultural production.

**Conclusions:**

Development of an improved genome assembly for *N. tabacum* enables what we believe to be the first successful map-based gene discovery for the species, and demonstrates the value of an improved assembly for future research in this model and commercially-important species.

**Electronic supplementary material:**

The online version of this article (doi:10.1186/s12864-017-3791-6) contains supplementary material, which is available to authorized users.

## Background

As the first plant to be adapted for tissue culture and among the first to be genetically engineered [1, 2], tobacco made two key contributions to molecular plant biology. More recently, potential applications in biopharmaceutical [3, 4] and biofuel [5] production have generated renewed interest in the species. Improved tobacco genomic resources are necessary to facilitate such applications, but genome size and complexity has slowed their development.

Polyploidisation has occurred during the evolutionary history of the majority of flowering plants, suggesting a possible role in conferring selective advantages [6]. Such events are highly prevalent in the *Nicotiana* genus [7], including the relatively young allotetraploid *N. tabacum* (2n = 4 × = 48), which arose less than 0.2 Ma ago through the hybridisation of the ancestral parents *N. sylvestris* (2n = 24; maternal S genome donor) and *N. tomentosiformis* (2n = 24; paternal T genome donor) [8–10].

Efforts such as the Tobacco Genome Initiative (TGI) provided sequence data for a low coverage of Bacterial Artificial Chromosomes (BACs) and active parts of the *N. tabacum* genome (Gene-space Sequence Reads [GSRs]), which allowed for genome-scale characterisation of gene families such as transcription factors [11]. Similarly, the TGI and other efforts generated Expressed Sequence Tags (ESTs), which provided insight into the gene content of *N. tabacum* and facilitated studies of gene expression in the species [12]. However, development of more complete genomic resources was constrained by the relatively high cost and low output of traditional sequencing methods. The emergence of Next Generation Sequencing (NGS) technologies over the past-decade (reviewed in [13]) has reduced these barriers and made sequencing efforts in species with complex genomes like *N. tabacum* more feasible.

The tobacco genome is estimated to be approximately 4.5Gb in size [7, 14, 15], which is smaller than the combined estimated sizes of the *N. sylvestris* and *N. tomentosiformis* genomes (2.6Gb and 2.7Gb respectively [14]). A low coverage of NGS data was sufficient to demonstrate that this reduction in size was due to the preferential loss of repetitive sequence from the T-genome of tobacco [16]. More recently draft genome sequences were made available for the species as well as its ancestral parents [15, 17], which represented a significant step forwards for research in the plant. Although the assemblies currently available for three different cultivars of *N. tabacum* [15] provide a reasonable level of coverage, their utility is limited by less than 20% of the genome being anchored onto pseudomolecules.

As with many other crop species, tobacco can be categorized into multiple market classes that are differentiated by area of cultivation, agronomy, harvesting and curing methodologies, as well as plant genetics. The burley market class is one of the major classes of tobacco, and is characterised by a high degree of chlorophyll deficiency that is most evident on the stems, stalks, and leaf mid-veins. This trait is conferred by a double homozygous recessive genotype at the *Yellow Burley 1* (*YB1*) and *Yellow Burley 2* (*YB2*) loci [18–20], which were recently mapped to locations on tobacco linkage groups 5 and 24 [21].

In addition to the chlorophyll deficiency, mutations at the *YB* loci have also been shown to confer changes to tobacco leaf chemistry [22], including increased alkaloid levels and leaf nitrate nitrogen (NO_3_-N) that together likely contribute to the higher level of Tobacco Specific Nitrosamine (TSNA) class of toxicants associated with these plants [23]. The *YB* loci also confer reduced Nitrogen Utilization Efficiency (NUtE) and Nitrogen Use Efficiency (NUE) [23], which is consistent with recommended Nitrogen fertilizer application rates being considerably higher for burley tobaccos (168 to 308 kg/ha [24]) compared to other tobacco classes, such as the Virginia market class (56 to 90 kg/ha [25]).

Improving uptake and utilisation of Nitrogen by plants represents a significant challenge for sustainable agricultural production in future, given needs to increase agricultural production to feed a growing world population in the face of increasing fertilizer costs and a need to reduce environmental externalities associated with agrochemical use [26]. Here we present an improved *de novo* genome assembly for *N. tabacum* and use it to map-based clone the *YB* loci, identifying a pair of homeologous genes that may have implications for understanding and improving NUtE and NUE in tobacco as well as other crop plants.

## Results and discussion

### An improved genome assembly for tobacco

**Fig. 1:**
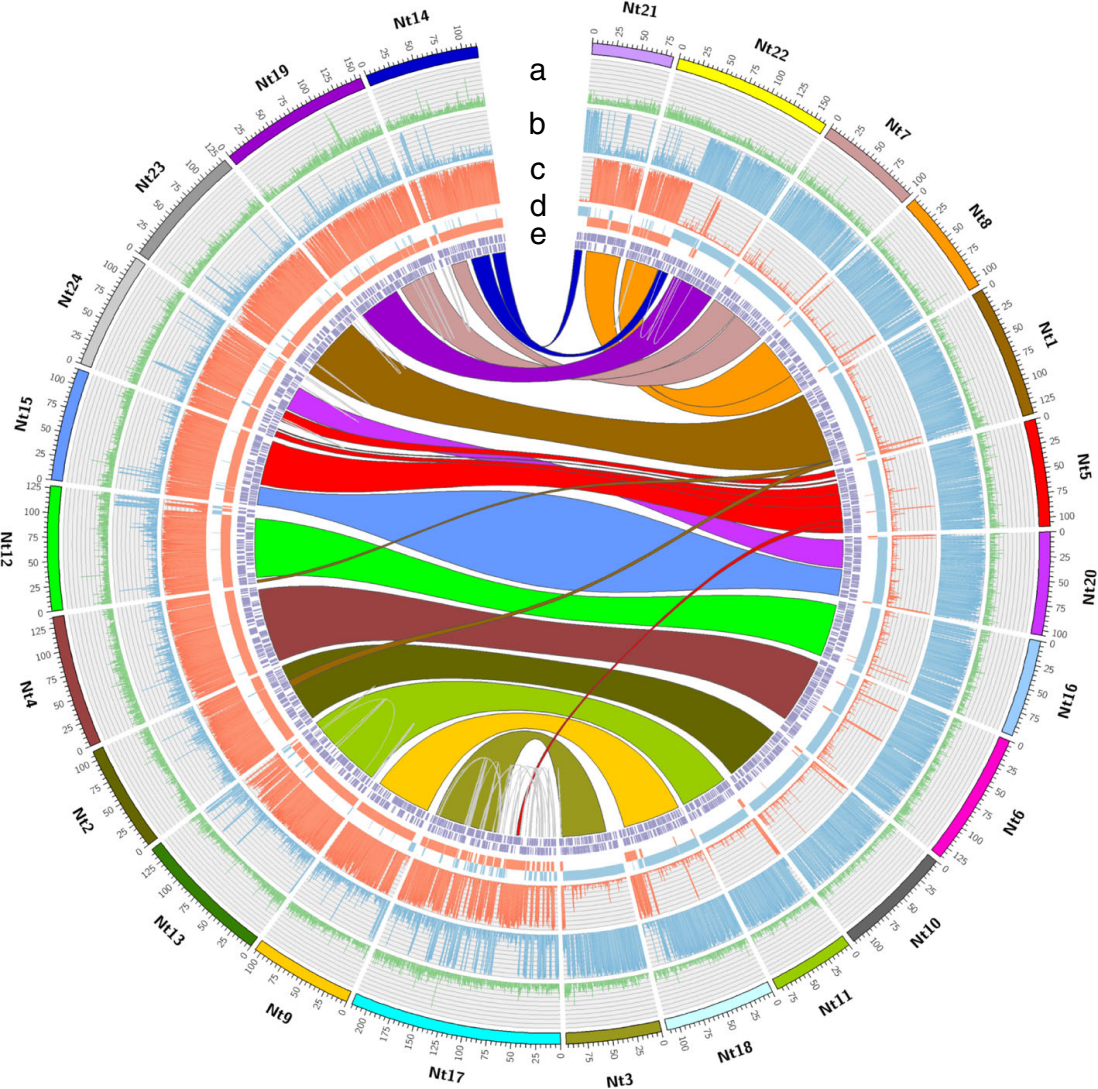
The tobacco genome. Circos plot showing the 24 pseudomolecules (Nt1–Nt24) generated by the tobacco genome assembly. With tracks for (**a**) gene density, (**b**) *N. sylvestris* sequence coverage, (**c**) *N. tomentosiformis* coverage, (**d**) regions of T- (*red bars*; *inner track*) or S- (*blue bars*; *outer track*) putative genome origin and (**e**) Physical super-scaffolds generated by hybrid assembly of NGS and optical map data anchored to the genetic map. Note that track e is split over two levels due to the density of the super-scaffolds visible at the displayed scale. Synteny between pseudomolecules is represented by *coloured linkers* across the centre of the plot. Tracks **a**, **b** and **c** represent density over 50 kb bins

**Fig. 2:**
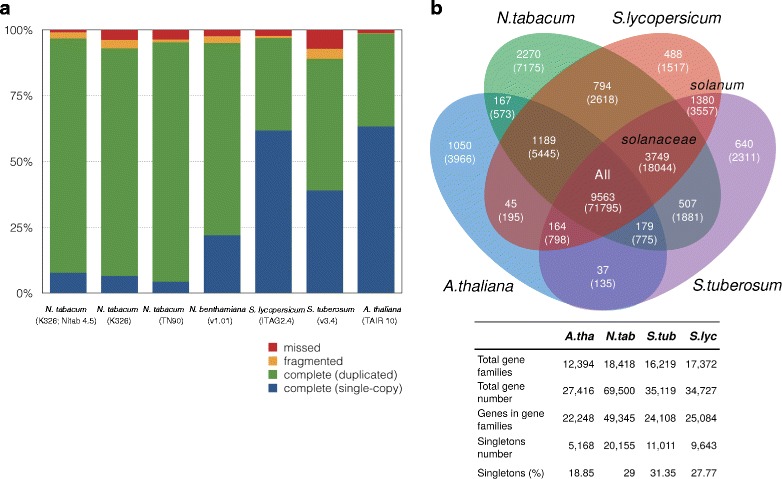
Tobacco Gene space (**a**) Analysis of completeness of the tobacco genome assembly versus other plant genome assemblies based on mapping of a set of universal single-copy orthologs using BUSCO [27]. Bar charts showing missing- (*red*), fragmented- (amber), complete duplicated- (*green*) and complete single-copy genes (*blue*) shown for the presented assembly (*N. tabacum* K326), along with the previously published *N. tabacum* assemblies for cultivars K326 and TN90 [15], *N. benthamiana* [66] tomato (ITAG2.4), potato (v3.4) and Arabidopsis (TAIR10). **b** Venn diagram showing the cross-over of gene families between tobacco (*N. tabacum*; *green*), tomato (*Solanum lycopersicum*; *red*), potato (*S. tuberosum*; *purple*) and Arabidopsis (*A. thaliana*; *blue*). Number of gene families is show for each intersection, with number of individual genes contained within each set shown below in parentheses. Table summarising the number of genes and gene families within each species

In order to improve the genomic resources available for *N. tabacum* we have generated a new Next Generation Sequencing (NGS) assembly for the species (version Nitab4.5; Fig. 1). The assembly covers over 4Gb of non-N sequence (90% of predicted genome size; Table 1), which is an increase from 3.6Gb (81% of predicted genome size) in the previously published version [15]. Analysis of gene content coverage [27] showed lower levels of missing and fragmented sequences compared to the previously available tobacco genome assemblies (Fig. 2a), which together with the increased assembly size (Additional file 1) suggests that it provides more complete coverage of the tobacco genome. Furthermore, the low level of missed or fragmented single-copy orthologs in the *N. tabacum* assembly is comparable to, or lower than the level shown by the tomato and potato genome assemblies respectively (Fig. 2a), supporting the quality of this current genome assembly for tobacco. User-friendly access to the *N. tabacum* assembly is available via the Solanaceae Genomics Network (SGN [28]; https://solgenomics.net).

**Table 1:** Tobacco Genome Assembly Statistics

	Nitab4.5 NGS assembly	S sub-genome	T sub-genome	BioNano Optical Map	Hybrid assembly	Pseudo-molecules
Number of contigs/scaffolds	1,084,432	386,863	228,210	3945	2217	24
Total length (Mb)	4695	2418	1859	3932	3688	2924
Total defined bases (Mb)	4049	1997	1544	–	–	1742
Max length (Mb)	5.99	2.21	5.99	9.04	13.67	216
N_50_ length (Mb)	0.28	0.21	0.66	1.33	2.17	116
Anchored sequence (Mb)	2924	715	1124			

Table showing assembly statistics for the different levels of the tobacco genome assembly. Statistics for the S and T sub-genomes are based on calling ancestral origin of Nitab4.5 NGS assembly following mapping of sequence reads from *N. sylvestris* and *N. tomentosiformis* [17]

Genome annotation identified repeat families covering 67% of the assembly (Additional file 2), and predicted 69,500 genes with structures comparable to genes from other *Solanaceae* (Table 2). Gene family and Gene Ontology analysis also showed good cross-over with the related *Solanaceae* species tomato and potato, in addition to other flowering plants (Fig. 2b and Additional files 3 and 4).

**Table 2:** Comparison of gene numbers and structures across the *Solanaceae*

	Number of genes	Average length (bp)	Average number exons	Average exon length (bp)	Average intron length (bp)
*N. tabacum*	69,500	4581.12	4.79	230.26	918.56
*N. benthamiana*	59,814	5141.92	5.05	273.96	926.26
*N. sylvestris*	37,162	4424.26	4.71	306.37	802.04
*N. tomentosiformis*	36,509	4151.92	4.69	309.17	729.81
*S. lycopersicum*	34,763	3163.36	4.61	262.38	541.97
*N. tabacum/* *S. lycopersicum*	2	1.45	1.04	0.88	1.69

Table showing statistics for predicted gene models from *N. tabacum* assembly as well as other *Nicotiana* species and tomato (*S. lycopersicum*; iTAG v2.30). Data for other *Nicotiana* species based early access to the *N. benthamiana* v1.0.1 assembly and re-annotations of the *N. sylvestris* and *N. tomentosiformis* genomes [17] using Maker and the publicly available transcriptomic data provided by the *N. benthamiana* genome sequencing consortium

### Evolution of the tobacco genome

**Fig. 3:**
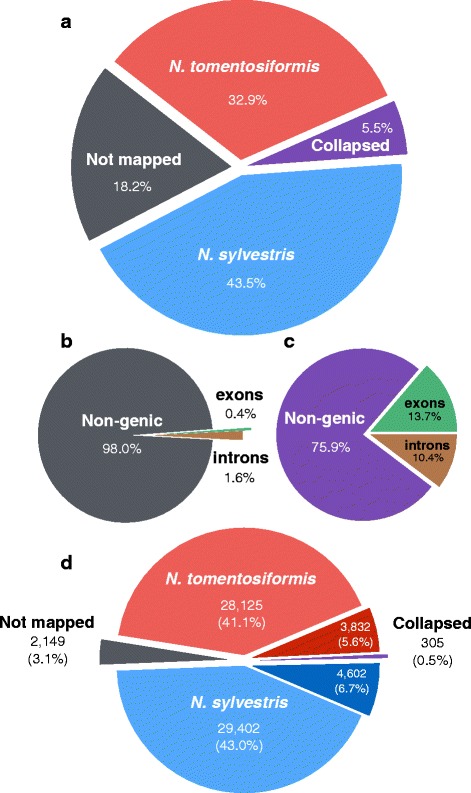
Ancestral origin of the tobacco genome (**a**) pie chart showing percentage of the tobacco genome assembly that is mapped by sequence reads from *N. tomentosiformis* (*red*) and *N. sylvestris* (*blue*), neither species (Not mapped; *grey*), or both species (Collapsed; *purple*). **b**, pie chart showing percentage of the Not mapped regions of the tobacco genome from (**a**) that are contained in Non-genic sequence (*grey*), exons (*green*), or introns (*orange*). **c**, pie chart showing percentage of the Collapsed regions of the tobacco genome from (**a**) that are contained in Non-genic sequence (*purple*), exons (*green*), or introns (*orange*). **d**, Number of genes (with percentage of total genes shown below in parentheses) that could be assigned to *N. tomentosiformis* (*red*) and *N. sylvestris* (*blue*) origin, or were not mapped (*grey*) or mapped by both species (Collapsed; *purple*) displayed. Genes in the collapsed set that could be putatively assigned to *N. tomentosiformis* (dark red), or *N. sylvestris* (*dark blue*) origin based on conserved sequence polymorphisms are also shown

Mapping of sequence reads generated from *N. sylvestris* and *N. tomentosiformis* [17] covered more than 80% of the tobacco genome assembly, which allowed the ancestral origin of much of the sequence to be established (Fig. 3a). Mapping of reads from *N. otophora* [15], which has also been proposed as a potential paternal genome donor for *N. tabacum* [8–10, 15], only covered 22.9% of the genome assembly, and these reads showed a tendency to be located in the same regions as *N. tomentosformis* reads, which supports *N. tomentosiformis* as the paternal genome donor, given the higher level of genome coverage shown by this species. The parts of the genome assembly that were unmapped by either *N. sylvestris* or *N. tomentosiformis* reads (18.2% of the assembly; Fig. 3a) may partially be explained by introgressions from other *Nicotiana* species introduced during commercial breeding for disease resistance in modern cultivars. For example, *N. otophora* reads mapped to just over one tenth of the of the 18.2% of the genome that was not mapped by *N. sylvestris* or *N. tomentosiformis* reads, which supports the possibility that such parts of the genome may originate from other *Nicotiana* species. However, approximately 98% of the unmapped regions were located outside of gene space (Fig. 3b), which was significantly greater than might be expected relative to the whole assembly (*p* < 5 × 10^−16^ Chi-squared test) and may be due to lower selective pressures, resulting in more rapid divergence from the ancestral sequence in these regions.

Our results demonstrate a reduced contribution of *N. tomentosiformis* to the tobacco genome (Fig. 3a); consistent with the loss of repetitive sequence from the T-genome [16]. Interestingly, this reduction in repetitive sequence makes the T-genome of *N. tabacum* easier to assemble as demonstrated by assembly statistics for the two sub-genomes (Table 1).

Reads from both ancestral species mapped equally well to 5.5% of the assembly, suggesting potential collapse of the assembly at these locations, or sequence loss from one parental genome during the evolution of tobacco (Fig. 3a). Approximately 24% of this sequence was present in gene space (Fig. 3c); significantly higher than would be expected by chance (*p* < 5 × 10^−16^ Chi-squared test). Polymorphisms between the *N. sylvestris* and *N. tomentosiformis* sequences allowed the majority of these genes to be putatively assigned to an ancestral genome, supporting the suggestion that the homeologous gene from the other ancestral parent has been lost in these 8434 cases (Fig. 3d). This low level of putative gene loss is consistent with the high number of predicted genes identified (69,500) compared to other *Solanaceae*, and the combined total of predicted genes from the *N. sylvestris* and *N. tomentosiformis* genomes (73,671; Table 2). A low level of gene loss is also consistent with the relatively high number of duplicated copies of proposed single-copy orthologs identified in the tobacco genome assembly compared with other sequenced diploid plant species such as tomato, potato and Arabidopsis (Fig. 2a).

Unlike in the inter-genic sequence, our analysis showed no evidence for preferential loss of genes from either ancestral parent (Fig. 3d). Transcriptomic analysis of three different tissues showed evidence of expression for a majority of predicted genes (50,665), with 42,487 expressed in all conditions (Additional file 5). This suggests that, in the majority of cases, both T- and S-genome copies of homeologous genes have not only been maintained, but are also expressed. Our previous research of transcriptomic data showed limited evidence for neo-functionalisation in *N. tabacum* [29]. This indicates that a high level of redundancy is present between *N. tabacum* genes, and suggests that this species may be too young in evolutionary terms for mechanisms such as gene-loss or neo-functionalisation to have contributed broadly to the shape of the tobacco genome through natural- and domestication-based selective pressures. Consistent with this, the *N. benthamiana* genome, which has had a longer period of time since the polyploidization event that formed the species for mechanisms such as gene loss to occur [30], shows a higher number of single-copy genes compared to *N. tabacum* (Fig. 2a).

### Organisation of the tobacco genome

Whole genome physical maps have been shown to provide invaluable frameworks for scaffolding NGS assemblies [31]. Using a BioNano Genomics optical map for tobacco we were able to consolidate 3.7Gb of the assembly onto 2217 scaffolds with an N_50_ size of 2.17 Mb; nearly an 8-fold increase from the 0.28 Mb N_50_ size of the NGS assembly alone (Table 1). This facilitated anchoring of approximately 64% (2.9 Gb) of the tobacco genome into pseudomolecules based on their locations on a high-density consensus genetic map (Table 1 and Fig. 1). The 24 pseudomolecules represented the diploid number of chromosomes of *N. tabacum* and, with the exception of Chromosome Nt17, showed good separation based upon T- and S-origins (Fig. 1d). The results of Chromosome Nt17 may be due to varying introgressions or lineage specific chromosomal rearrangements [32] in this group.

Based on synteny, seven pairs of homeologous chromosomes could be clearly identified (Fig. 1). Chromosomes Nt5, Nt15, Nt20 and Nt24 exhibited more complex relationships, which indicated that further rearrangements may have occurred between chromosomes of the *N. sylvestris* and *N. tomentosiformis* genomes, either before or after the formation of *N. tabacum* (Fig. 1). Additional intra-genome rearrangements appear to have occurred based upon synteny between Nt7 and both Nt19 and Nt14, which also exhibit synteny with regions of *N. sylvestris* origin on Nt21 and Nt22 (Fig. 1). However, both Nt21 and Nt22 appear to contain large blocks of sequence of *N. tomentosiformis* origin, which exhibit synteny with chromosome Nt8 of *N. sylvestris* origin, suggesting that an inter-genome rearrangement has occurred between these set of chromosomes (Fig. 1). A further rearrangement is indicated at the end of Nt18, which exhibits a block of *N. tomentosiformis* sequence in a chromosome of predominantly *N. sylvestris* origin, and a small cross over in the opposite direction at the end of Nt9, which is supported by previous results [15], suggesting a reciprocal cross-over between these chromosomes (Fig. 1). These observations are consistent with the proposed number of inter-genome recombinant chromosomes present in cultivated tobacco based on previous genomic *in situ* hybridisation (GISH) analysis [32].

### Genetic differentiation of burley tobacco

**Fig. 4:**
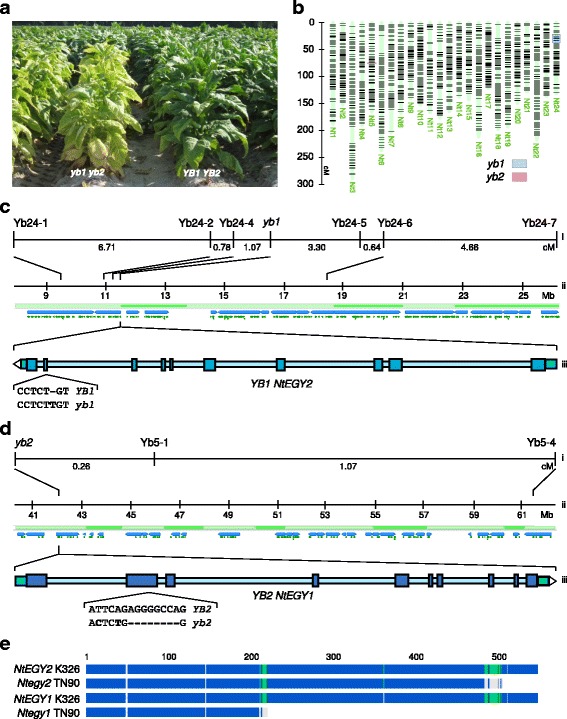
Map-based cloning of the *yb* mutant genes *NtEGY1* and *NtEGY2*. **a** picture showing yellow, chlorotic phenotype of *yb1 yb2* genotype NIL (*left*) versus wild type *YB1 YB2* parent (*right*) in one of the lines used in mapping of *yb* loci (Cultivar SC58). **b**, High density genetic map for tobacco (*N. tabacum* 30 k Infinium HD consensus map 2015; https://solgenomics.net/cview/map.pl?map_version_id=178) showing location of SNP markers linked to *yb1* (*blue box*) on *Nt24* and *yb2* (*red box*) on *Nt5*. Mapping of *yb1* (**c**) and *yb2* (**d**) loci showing position of SNP markers linked to the loci on (i) genetic and (ii) physical maps. Physical map shows position of super-scaffolds (*alternating light and dark green bars*) and underlying sequence scaffolds/contigs (*blue bars*), as well as genes (*green triangles*). Position of *NtEGY1* and *NtEGY2* in physical map shown (iii) with schematic representation of exons (*wide dark blue boxes*), introns (*narrow light blue bar*) and 5’ and 3’ UTRs (*intermediate blue boxes*), with direction of gene indicated by *white arrow-head* at 3’end. Sequence polymorphisms between wild type and mutant alleles indicated, showing single base insertion in exon 9 of *NtEGY2* (**c**) and 8 bp deletion in exon 2 of *NtEGY1* (**d**). **e**, protein alignment based on predicted sequence translated from cDNA of NtEGY1 and NtEGY2 from *YB1 YB2* genotype K326 and *yb1 yb2* genotype TN90 cultivars, showing truncated proteins produced from the TN90 alleles of the genes. *Coloured regions* of alignment indicate sequence identity between the four proteins (*dark blue* 100%, *green* 60–80%, and *grey* <60%)

Tobacco can be categorized into multiple market classes. The K326 cultivar sequenced in this study is of the Virginia market class, the widest-grown class. Varieties of burley market class tobacco exhibit a strong chlorophyll deficient phenotype (Fig. 4a), known to be conferred by a double homozygous recessive genotype at the *YB1* and *YB2* loci [18–20], which have also been associated with other plant physiology and leaf chemistry traits [22]. The *yb1 yb2* genotype was recently shown to cause reduced nitrogen utilization efficiency, and increase levels of alkaloids and leaf nitrate (NO_3_-N) [23], likely contributing factors to higher levels of carcinogenic TSNA compounds typically found in this market class [23].

A combination of high levels of redundancy between genes in such a large and complex genome, together with a historical absence of molecular markers and genomic resources, has made identification and subsequent mapping of interesting mutants a very difficult prospect in tobacco. However, having anchored 64% of the genome assembly to chromosomal locations, a possibility now exists to apply map-based gene discovery approaches in the species.

### Map-based cloning of the *YB* loci

Genotypic analysis of three previously described pairs of nearly isogenic lines (NILs), differing from their respective parents at genomic regions carrying *YB1* and *YB2* loci, [23] resulted in the identification of two genomic regions that consistently distinguished the NILs from their isogenic parents (Fig. 4b). In agreement with previous mapping efforts [21], these loci were located on chromosomes Nt5 and Nt24, which were donated by *N. sylvestris* and *N. tomentosiformis,* respectively. Given the proposed ancestral origin of the *YB* loci [19], this suggested that *YB1* and *YB2* are represented by genes present on Nt24 and Nt5, respectively. To confirm linkage and support fine mapping of *yb1* and *yb2*, several markers across the two loci were assayed in two mapping populations designed to be homozygous recessive for one locus and segregating for the other. This located *yb1* to within a 4.37 centi-Morgan (cM) interval between SNP markers Yb24-4 and Yb24-5, and *yb2* within 0.26 cM of SNP marker Yb5-1 (Fig. 4c and d).

Earlier characterisation of the *yb* loci suggested that their genetic effects were manifested in the leaves rather than the root [33, 34], and it has been speculated that the difference may be related to chlorophyll synthesis; with lower levels of precursors and higher chlorophyllase activity shown in burley cultivars [35]. It could also be speculated that genes involved in nitrogen assimilation or transport might underlie the observed chlorophyll deficiency of burley tobacco plants, although previous analysis indicated few differences in gene expression in major nitrogen assimilation genes in burley tobacco [15]. Linking the genetic map to the genome assembly allowed the identification of genes in the vicinity of the two loci (Fig. 4c and d). Genes predicted to be associated with nitrogen assimilation, nitrogen use physiology, or chloroplast activities were prioritised for analysis and a comparison was made of their sequence from this study (variety K326; *YB1 YB2* genotype) to those from the previously sequenced *yb1 yb2* burley cultivar TN90 [15]. Homologs of *Arabidopsis thaliana ETHYLENE-DEPENDENT GRAVITROPISM-DEFICIENT AND YELLOW-GREEN 1* (*AtEGY1*) were identified as strong candidate genes (hereafter called *NtEGY1* and *NtEGY2*), as they contained sequence polymorphisms predicted to result in truncated proteins in the TN90 alleles (Fig. 4c, d and e).

Manual annotation of the gene models indicated a gap in the sequence of *NtEGY1*, which affected the end of Exon 1 and start of Exon 2. To fill in this gap and validate the predicted gene models and sequence polymorphisms, cDNA sequences were generated from RNA of the K326 and TN90 cultivars (Additional file 6). Predicted *NtEGY1* and *NtEGY2* proteins shared 97.98% identity, and both proteins showed strong alignment to *AtEGY1* (73.23 and 73.65% identity respectively) as well as with related proteins from other plant species, suggesting that these genes are members of the chloroplast-targeted MEROPS M50 family [36] (Additional file 7). This analysis also confirmed the ancestral origin of *NtEGY1* (*N. sylvestris*) and *NtEGY2* (*N. tomentosiformis*). Together with synteny between chromosomes Nt5 and Nt24 (Fig. 1a) this strongly suggested *NtEGY1* and *NtEGY2* to be homeologs.

Consistent with *NtEGY1* and *NtEGY2* being homologs of the Arabidopsis gene *AtEGY1*, their expression patterns were similar to *AtEGY1*, with high expression levels in the shoot apex and leaves, and low levels in the root (Additional file 8). This expression pattern is also consistent with the *yb* phenotype being manifested from the leaf [33, 34]. *AtEGY1* encodes a membrane-bound, plastid-targeted, and ATP-independent metalloprotease site-2 protease that is required for development of thylakoid grana, a well-organized lamellae system, and accumulation of chlorophyll and chlorophyll a/b binding proteins in chloroplast membranes [36]. *Arabidopsis egy1* mutants exhibit a yellow-green plant phenotype [36, 37] similar to the observed chlorotic phenotype in *yb1 yb2* genotype tobacco plants (Fig. 4a). However, whereas in *Arabidopsis* this phenotype is visible throughout plant development, *yb1 yb2* double mutants are generally not distinguishable from wild-type individuals until about 40 days after germination. This is more similar to the phenotype shown in mutants for the tomato *EGY1*-like gene, *lutescent2* (*l2*), which results in an enhanced rate of chlorophyll loss in leaves and fruits as plants mature [38].

Genotypic markers specific to *NtEGY1* and *NtEGY2* alleles exhibited complete linkage with the chlorophyll-deficient phenotype in more than 1000 individuals from the mapping populations described above. Twelve additional white-stemmed burley cultivars tested also carried these alleles, while other market classes showed genotypes consistent with these genes underlying the *yb* phenotype and the differentiation of the burley market class (Additional file 9).

## Conclusions

Improving the tobacco genome assembly, and in particular increasing the anchorage of the tobacco genome to chromosomal locations from 19% [15] to 64%, has provided a genomic roadmap that will help serve acceleration of many aspects of tobacco and plant science research. Although further improvements to the assembly would be desirable, the current research represents a substantial step forward, with the benefits of optical mapping for improving the coherency of assemblies in such complex genomes being readily apparent. Using the assembly, we were able to map-based clone *NtEGY1* and *NtEGY2. * Mutations in these genes have been selected for during the domestication and breeding of an entire market class of tobacco. We believe that this represents the first reported instance of successful map-based cloning in tobacco, and indeed one of the few successful examples alongside wheat [39] of this approach being used in species with large polyploid genomes. The added complexity of mapping mutants in polyploid species - particularly highly redundant ones such as tobacco - is well demonstrated by the requirement for mutations in both homeologous genes *NtEGY1* and *NtEGY2* to uncover the recessive *yb* mutant phenotype. Modification of these genes could have implications for management of TSNA toxicants in tobacco, as well as for improving nitrogen utilization efficiency in plants and contributing towards more sustainable agricultural production.

## Methods

### Tobacco genome sequencing and assembly

#### Whole genome sequencing (WGS)

A whole genome shotgun sequencing approach was taken to sequence the tobacco genome. Genomic DNA was extracted from young leaves of 7 week old tobacco seedlings (var. K326; PI552505) using Qiagen DNeasy Plant Mini Kits (Qiagen, Hilden, Germany). Shotgun and Paired End Roche 454 data was generated using GS FLX Titanium and FLX+ chemistry (Roche 454, Branford, CT). Illumina Paired End (PE) and Mate Pair (MP) data was generated on a HiSeq 2000 (Illumina, San Diego, CA) at the DNA Sequencing unit of the Cornell University Core Laboratories Center according to manufacturer’s instructions. Additional file 10 summarises the data generated from each library type included in the assembly.

GS FLX Titanium data representing approximately 17× depth of the tobacco genome was assembled using gsAssembler v2.8 (Roche454, Branford, CT) using default settings with the exception of minimum identity and minimum overlap length being set to 97% and 100 bp respectively. This assembly was corrected for sequence errors, including homopolymers, by mapping the 300 bp Illumina PE sequence to the assembly, calling the SNPs, filtering them based on the read coverage and the allele frequency and applying the Perl script Vcf4FastaCorrection (available from https://github.com/aubombarely/GenoToolBox/blob/master/SeqTools/Vcf4FastaCorrection). The resulting WGS assembly contained 440,772 contigs/scaffolds and covered 4,145,428,719 bp (3,510,246,061 bp of which were defined), with an N_50_ of 334,966 bp.

#### Whole genome profiling (WGP)

In addition to the WGS approach, local assemblies were generated based on sequence tags from Whole Genome Profiling (WGP) of Bacterial Artificial Chromosomes (BACs) by KeyGene (Wageningen, Netherlands). Two libraries containing 150,528 BACs each were generated by Amplicon Express (Pullman, WA, USA) using *Hind*III or *Eco*RI, with average insert sizes of 115 kb and 135 kb respectively (representing approximately 8× coverage of the tobacco genome). A WGP map was generated from sequence reads at *Eco*RI and *Hind*III restriction sites as described previously [40] with the exception that Illumuina PE 100 bp reads were used in the analysis instead of single end reads. The WGP map was divided into 369,215 bins based on BAC overlaps, in which each bin represented a distinct part of a BAC contig or singleton BAC in the WGP assembly. 1,715,071,552 filtered unique read pairs were assigned to local bins and assembled using PHRAP into 9,499,445 contigs with an N_50_ length of 693 bp. Sequence contigs that were generated from different bins on the same WGP BAC contig were subsequently assembled into 3,989,136 contigs with an N_50_ length of 819 bp covering a total of 3.3Gb.

To align the WGS and WGP assemblies, BLAST searches of the WGP contigs were carried out against the WGS assembly. High Scoring Pairs (HSPs) identified were used to extend or fill gaps in the contigs/scaffolds from the WGS assembly where equal to or greater than 95% sequence similarity was shown over the HSPs and the gaps/extensions were less than 10 bp or less than 10% of the total length of WGP contigs. In cases where the same region of individual WGP contigs matched multiple WGS contigs/scaffolds, the best matching WGS contig/scaffold was selected based on highest HSP length coverage (where the ratio was greater than 0.6 compared to alternative matches and providing co-linearity of HSP order was preserved between the sequences). If multiple WGP contigs matched the same region of a WGS contig/scaffold and was flanking a gap, then the same criteria was used to select the matching sequence. In cases where multiple WGP contigs matched to the same WGS contig/scaffold and did not flank a gap, then the best matching local contig was selected based on the one showing highest HSP length coverage and shortest overhang. The remaining local contigs that did not meet these criteria were appended to the assembly resulting in an integrated NGS assembly with a total number of 1,093,289 contigs/scaffolds covering 4,675,833,176 bp (4,052,946,448 defined bp) with an N_50_ length of 268,762 bp.

### Scaffolding and gap-filling

The NGS assembly was further scaffolded using SSPACE [41] v2.2 (Baseclear, Leiden, Netherlands) with default parameters. Reads were mapped to the assembly using bowtie2 [42] v2.0.6 and reformatted into TAB format as input to SSPACE in the order of: 300 bp PE (Illumina, San Diego, CA), 2 k MP (Illumina), 2 k PE (454), 7 k PE (454), 8 k MP (Illumina, San Diego, CA), and 20 k PE (454). Gaps were filled in the subsequent assembly using Gapfiller [43] v1-10 (Baseclear, Leiden, Netherlands) with default parameters and the 300 bp PE Illumina reads, to produce the final NGS assembly (Nitab4.5). Gapfiller was set to run for 10 iterations, but was stopped after 8 as further iterations were not making any additional improvement to the assembly. All of the reads generated and used in the assembly process are available from the National Center for Biotechnology Information (NCBI) Short Read Archive (SRA), associated with study SRP100451. The final version of the NGS assembly is available from the SGN (https://solgenomics.net/). A version of the assembly is also available from the NCBI (https://www.ncbi.nlm.nih.gov/bioproject/?term=PRJNA376174), following filtering of scaffolds shorter than 500 bp and according to NCBI requirements.

### Construction of genome maps using the Irys system for contig anchoring and scaffolding

Optical map generation and scaffolding was carried out by BioNano Genomics (BNG; San Diego, CA). High molecular weight (HMW) genomic DNA was isolated from tobacco leaves using the following protocol outline. 2.5 g of frozen young leaf tissue was fixed with 2% formaldehyde. After washing out the formaldehyde with isolation buffer, blending with a tissue homogenizer was performed. Triton-X treatment was used to release nuclei from the cells. The nuclei were purified on Percoll cushions, washed, and embedded into low melting point (LMP) agarose gel plugs at different dilutions. Finally, the DNA plugs were treated with a lysis buffer containing detergent and proteinase K. β-mercaptoethanol (BME) was used throughout the entire prep (through proteinase K treatment) to prevent oxidation. Gel plugs were treated with RNase, washed, melted, solubilized, and dialyzed. Resulting HMW DNA was fluorescently labelled with nickase Nt.BspQI using the IrysPrep kit. A total of 575 Gb of molecules (filtered by molecule length >150 kb) were collected on the Irys system, representing ~110X genome coverage with a molecule length N_50_ of 278 kb.

#### De novo assembly

The BNG genome maps were generated with RefAligner [44, 45] assembler (v3686) and assembly pipeline (v3728) using DNA molecules images from Irys (BioNano Genomics). With default pipeline parameters (optArguments_medium.xml), a draft genome assembly was generated and used as a reference to run the final assembly. This second assembly was used in hybrid scaffolding with the NGS assembly. 3945 genome maps were assembled with an N_50_ of 1.35 Mb spanning 4.01 Gb. Associated .bnx and .cmap files are available from the NCBI via BioProject PRJNA376174 (also available from https://submit.ncbi.nlm.nih.gov/ft/byid/GrjhypUE/K326_exp_refineFinal1_contigs.cmap and https://submit.ncbi.nlm.nih.gov/ft/byid/vyb7psJM/Molecules.bnx respectively).

### Hybrid scaffold generation

The hybrid scaffolding of BioNano genome maps and NGS contigs were carried out using BioNano‘s scaffolding pipeline NGM Hybrid Scaffold (NGM-HS) (version 3632) and alignment tool RefAligner [44, 45]. First, an *in silico* map of BspQI was generated from the NGS sequence contigs. Genome maps were aligned and merged with RefAligner using a threshold *P* value of 1 × 10^−10^, a minimum 50 kb alignment, and a minimum of 5 BspQI sites to create hybrid scaffolds [46]. The final set of hybrid scaffolds is 3.69 Gb in length and 2.17 Mb in genome map N_50_, representing an N_50_ improvement in contiguity of 7.75 fold. To maximize the sequence content in the hybrid scaffolds, the sequence contigs were aligned to the hybrid scaffolds using a less stringent threshold *P* value (1 × 10^−8^). Lastly, NGM-HS outputs an AGP and a FASTA, which are available to download from the SGN (ftp://ftp.sgn.cornell.edu/genomes/Nicotiana_tabacum/edwards_et_al_2017/).

### Anchoring to genetic map

Genetic markers from the *N.tabacum* 30 k Infinium HD consensus map 2015 (https://solgenomics.net/cview/map.pl?map_version_id=178) were mapped to the Nitab4.5 NGS assembly and translated to the hybrid assembly based on inclusion of Nitab4.5 scaffolds in the hybrid assembly super-scaffolds. Super-scaffolds were assigned to linkage groups on the genetic map, named according to Bindler et al. [47]. Absolute orientations of super-scaffolds could not be determined, so were arbitrarily assigned, and the sequences were linked together by 100 Ns to form Pseudo-molecules Nt1 to Nt24.

### Benchmarking of assembly

The completeness of the tobacco assembly was assessed based on the representation of a universal set of single-copy orthologs using BUSCO [27]. Genome sequences for previous *N. tabacum* assemblies and other plant species were analysed for comparison as indicated.

## Gene model prediction

Tobacco gene models were predicted using Maker v2.28 [48] with Augustus [49] and SNAP [50] for *Ab-initio* gene model prediction. Tomato gene cDNA from iTAG v2.30, a collection tobacco of RNA-seq libraries from several tissues and conditions, and a selection of *Solanaceae* proteins and tobacco unigenes [29] were used as gene evidence reference. Intron-exon statistics for tobacco and tomato (iTAG v2.40) genomes were calculated using custom Perl scripts and the gene model gff files downloaded from SGN.

Functional annotation of gene models was carried out using InterProScan v5.2–45 [51] and InterPro database v 45.0, as well as BLAST searches against TAIR 10 [52], SwissProt [53], and iTAG 2.40, to assign GO terms and functional descriptions. iTAK software version 1.2x64 (http://bioinfo.bti.cornell.edu/cgi-bin/itak/index.cgi) was used to identify and classify protein kinases and transcription factors among the tobacco genes.

Gene Ontology (GO) analysis for tobacco, tomato, potato and Arabidopsis was performed with GOProfiles [54] at level 2 for Biological Process, Molecular Function, and Cellular Component terms. The tobacco GO terms were obtained from the InterProScan analysis, potato from Solanaceae Genomics Resource (SGR; http://solanaceae.plantbiology.msu.edu/), tomato from the SGN, and the GO terms for Arabidopsis from TAIR (v 10).

A TobaccoCyc metabolic pathway annotation was performed using a custom Perl script to create the pathologic input files from the annotation results based on BLAST searches of SwissProt. The TobaccoCyc (v1.0) metabolic pathway database was created using Pathway-tools v17.5 [55].

Gene family analysis was carried out using default parameters (e-value 1e^−5^ and inflation 1.5) in OrthoMCL software v2.0.9 [56] with protein sequences (without splice variants) from *Zea mays*, *Oryza sativa*, *Vitis vinifera*, *Arabidopsis thaliana*, *Glycine max*, *Populus trichocarpa* and *Solanum tuberosum* downloaded from Phytozome [57] and from *Solanum lycopersicum* downloaded from SGN [28].

## Analysis of repeat families

Identification of repeat sequences was carried out using Repeatmasker v4.0.6 with a specific database of repeats from the tobacco genome generated with RepeatModeler, together with a combination of plant repeats from RepBase including the Arabidopsis thaliana and the dicots repeat databases. The repeats database for RepeatMasker, repeatmaskerlibraries-20150807, was downloaded from Repbase, at the Genetic Information Research Institute website (http://www.girinst.org/).

## Assigning of ancestral origin to sequences

Sequence reads from *N. sylvestris* and *N. tomentosiformis* as described by Sierro et al. [17] were obtained from the Sequence Read Archive (SRA; NCBI) and mapped to the assembly using bowtie2 [42] filtering the output to allow only 5 mismatches. Bedtools and custom perl scripts were used to count the nucleotides from the sections “*N. tomentosiformis”*, “*N. sylvestris”*, “Not mapped” and “Overlapped”. Genes were assigned ancestral origin if at least half their length was covered by sequence reads from one ancestor with at least double the coverage depth of the other ancestor. Genes not meeting these criteria but showing a higher coverage and at least 10 reads greater depth than the other ancestor were classified as putative. Nitab4.5 scaffolds were classified as originated from one ancestor when this ancestor mapped more than 50% of the scaffold and the other ancestor less than 10%.

## Synteny analysis

MCScanX software [58] was use to find syntenic blocks based on collinear genes. For representation Circos v0.68-1 [59] was used to display a simplification where large blocks of collinear genes were merged onto wide ribbons.

## RNA-seq analysis

RNA was extracted from root, whole shoot, and shoot apex tissues from 8 week old, long-day (18 l/6 days) tobacco plants (cv. K326), harvested at ZT0, 6, 12 and 18 using QIAzol followed by DNase treatment and clean up using Qiagen Plant RNeasy kits, according to manufacturer’s instructions. Library preparation of random primed cDNA-libraries using proprietary methods and sequencing was performed by GATC Biotech (Konstanz, Germany). Sequencing data was generated on Illumina HiSeq2000 instruments in 100 bp single read mode. For gene expression analysis, all reads were quality checked using FastQC (https://www.bioinformatics.babraham.ac.uk/projects/fastqc/), trimmed for adapter sequences and poor quality bases (>Q30) using fastq-mcf (https://expressionanalysis.github.io/ea-utils/) with the following parameters *q = 30, l = 50* and *P = 30*. Reads were mapped against the predicted gene models and gene expression predicted using RSEM v1.2.7 [60]. RNA-seq quality metrics are presented in Additional file 5. Genes were considered as expressed in a tissue if transcripts per million (TPM) was ≥1 in each of the three biological replicates for at least one time-point. The data discussed in this publication have been deposited in NCBI’s Gene Expression Omnibus (GEO) [61] (accession number GSE95717; https://www.ncbi.nlm.nih.gov/geo/query/acc.cgi?acc=GSE95717).

## Map-based cloning of *yb1* and *yb2*

DNA was isolated from three previously described pairs of NILs carrying dominant or recessive alleles of the *YB1* and *YB2* loci (cultivars SC58, NC95, and Coker 1) [23] using a modified cetyltrimethylammonium bromide procedure [62]. DNA from these lines was genotyped with a custom 30 K Infinium iSelect HD BeadChip SNP chip (Illumina Inc., San Diego, CA) used in developing a high density genetic map (*N.tabacum* 30 k Infinium HD consensus map 2015; https://solgenomics.net/cview/map.pl?map_version_id=178). Genomic regions containing polymorphisms that differentiated the nearly isogenic lines were identified and corresponding SNP markers of interest were converted to Kompetitive Allele Specific PCR (KASP) markers [63] by LGC Genomics (Beverly, MA) (Additional file 11).

Doubled haploid DH lines BWDH8 (*yb1 YB2*) and BWDH16 (*YB1 yb2*) were produced by doubling of haploid plants according to Kasperbauer and Collins [64], generated from F_1_ hybrids of cultivars Ky14 (*yb1 yb2*) and K346 (*YB1 YB2*) pollinated by *N. africana*, according to Burk et al. [65]. For fine mapping of *yb1* and *yb2,* BC_1_F_1_ mapping populations were developed from F_1_ hybrids of these two DH lines crossed and then back-crossed to the homozygous *yb1 yb2* genotype burley tobacco breeding line NC1427-17. The two BC_1_F_1_ populations were expected to segregate at only one *yb* locus each, resulting in a 1:1 ratio for the yellow burley phenotype. Approximately 1000 of the BC_1_F_1_ progeny for each family were grown in a field at Clayton, NC, scored for the chlorophyll-deficient phenotype, and genotyped with KASP markers corresponding to SNPs found to be closely linked to either *YB* locus.

SNP markers found to be closely linked to the loci were aligned to the genome assembly and genes predicted to be involved in nitrogen assimilation, nitrogen use physiology, or chlorophyll maintenance were considered as potential candidates. Sequences for K326 (*YB1 YB2*) from this study and burley tobacco cultivar TN90 (*yb1 yb2*) from Sierro et al. [15] were investigated for polymorphisms in these candidate genes. Primers were designed to permit genotyping for polymorphisms of interest in *NtEGY1* and *NtEGY2* (Additional file 11) and tested in the previously described mapping populations to confirm linkage to the yellow burley phenotype.

## Isolation and cloning of *NtEGY1* and *NtEGY2* cDNA

RNA was extracted from leaf tissue of 6-week old plants of K326 and TN90 plants using the RNeasy Plant Mini Kit (Qiagen, Hilden, Germany). cDNA was synthesized using the SuperScript First-Strand Synthesis System for RT-PCR with oligo(dT) (Invitrogen, Carlsbad, CA). The coding regions of Yb candidate genes were amplified by PCR from first-strand cDNA from K326 and TN90 using the primers cYb-F and cYb-R (Additional file 11). Because few nucleotide differences existed between *NtEGY1* and *NtEGY2* at either the 5’ or 3’ ends, it was not possible to design primers specific to either homeolog. Bands were therefore excised from agarose gels and purified with the Monarch DNA Gel Extraction Kit (New England Biolabs, Ipswich, MA). Fragments were cloned into the pCR-Blunt vector using the Zero Blunt PCR Cloning Kit (Invitrogen, Carlsbad, CA) and transformed into NEB 5-alpha competent *E. coli* cells (New England Biolabs, Ipswich, MA). Sequencing of individual clones derived from each cultivar was carried out using vector primers. Sequences are available from NCBI (accession numbers KX507181- KX507184).

## Additional files

Additional file 1: Table showing assembly statistics for current genome assembly and previous publicly available release of tobacco genome. (PDF 259 kb)

Additional file 2: Table showing repeat families in *N. tabacum* genome. (PDF 241 kb)

Additional file 3: Gene family analysis of sequenced plant genomes. (PDF 455 kb)

Additional file 4: Bar charts and tables summarising functional annotation of tobacco gene models. (PDF 369 kb)

Additional file 5: Venn diagram showing numbers of tobacco gene models scored as expressed in root, shoot, and shoot apex samples. (PDF 420 kb)

Additional file 6: Alignment of cDNA sequence for *YB1* (*NtEGY2*) and *YB2* (*NtEGY1*) alleles from K326 and TN90 cultivars. (PDF 183 kb)

Additional file 7: Phylogenetic analysis of predicted protein sequences for *EGY1* homologs in various plant species. (PDF 303 kb)

Additional file 8: Expression pattern of EGY1 genes in tobacco and Arabidopsis. (PDF 234 kb)

Additional file 9: Table showing genotypic analysis of tobacco cultivars. (PDF 22 kb)

Additional file 10: Table summarising sequence data used in tobacco genome assembly. (PDF 289 kb)

Additional file 11: Tables summarising primer sequences used in cloning and genotyping analysis. (PDF 282 kb)

## Abbreviations

NGS: Next generation sequencing
NUE: Nitrogen use efficiency
NUtE: Nitrogen utilisation efficiency
SGN: Solanaceae genomics network
YB: Yellow burley
