# Supplementary material for: A reference genome for Nicotiana tabacum enables map-based cloning of homeologous loci implicated in nitrogen utilization efficiency
Source: BMC Genomics. 2017 Jun 19;18:448. doi: 10.1186/s12864-017-3791-6 (PMC5474855; doi:10.1186/s12864-017-3791-6)
Supplement: Supplementary file 1 — Table showing assembly statistics for current genome assembly and previous publicly available release of tobacco genome. (PDF 259 kb) [file 12864_2017_3791_MOESM1_ESM.pdf]

Supplementary Data 1 – Improvement of the tobacco genome assembly

	N.tabacum **(cv. K326) from this study**		***N. tabacum*** **(cv. TN90) from Sierro *et al.*, 2014**	
	**NGS**	**Super-Scaffolds**	**NGS**	**Super-Scaffolds**
**Number of contigs/scaffolds**	1084432	2217	829882	382373
**Total length (Mb)**	4695	3,688	3719	3719
**Defined bases (Mb)**	4049		3613	3613
**N50 length (Mb)**	0.28	2.17	-	0.39
**Anchored sequence**	-	2924	-	706

Comparison of genome assembly presented with previously best publicly available genome assembly for the cultivar TN90 (Sierro et al., 2014).
