# Supplementary material for: A reference genome for Nicotiana tabacum enables map-based cloning of homeologous loci implicated in nitrogen utilization efficiency
Source: BMC Genomics. 2017 Jun 19;18:448. doi: 10.1186/s12864-017-3791-6 (PMC5474855; doi:10.1186/s12864-017-3791-6)
Supplement: Supplementary file 2 — Table showing repeat families in N. tabacum genome. (PDF 241 kb) [file 12864_2017_3791_MOESM2_ESM.pdf]

Supplementary Data 2 - Table of repeat families identified in *N. tabacum* genome assembly

==
file name: Nitab4.5_scf.fasta
sequences: 1084432
total length: 4694948798 bp (4049313390 bp excl N/X-runs)
GC level: 38.88 %
bases masked: 3147747850 bp (67.05 %)
==

	number of elements*	length occupied	percentage of sequence
SINEs:	120259	18586975 bp	0.40 %
ALUs	35605	6801681 bp	0.14 %
MIRs	0	0 bp	0.00 %
LINEs:	179285	102406783 bp	2.18 %
LINE1	34825	31044780 bp	0.66 %
LINE2	5438	769720 bp	0.02 %
L3/CR1	0	0 bp	0.00 %
LTR elements:	2623836	2011947685 bp	42.85 %
ERVL	0	0 bp	0.00 %
ERVL-MaLRs	0	0 bp	0.00 %
ERV_classI	2571	1161543 bp	0.02 %
ERV_classII	3751	330136 bp	0.01 %
DNA elements:	388442	123764351 bp	2.64 %
hAT-Charlie	0	0 bp	0.00 %
TcMar-Tigger	0	0 bp	0.00 %
Unclassified:	2531206	863230291 bp	18.39 %
Total interspersed repeats:		3119936085 bp	66.45 %
Small RNA:	52972	7887422 bp	0.17 %
Satellites:	11836	11334626 bp	0.24 %
Simple repeats:	404843	25024792 bp	0.53 %
Low complexity:	76754	3815378 bp	0.08 %

==
