# Supplementary material for: A reference genome for Nicotiana tabacum enables map-based cloning of homeologous loci implicated in nitrogen utilization efficiency
Source: BMC Genomics. 2017 Jun 19;18:448. doi: 10.1186/s12864-017-3791-6 (PMC5474855; doi:10.1186/s12864-017-3791-6)
Supplement: Supplementary file 3 — Gene family analysis of sequenced plant genomes. (PDF 455 kb) [file 12864_2017_3791_MOESM3_ESM.pdf]

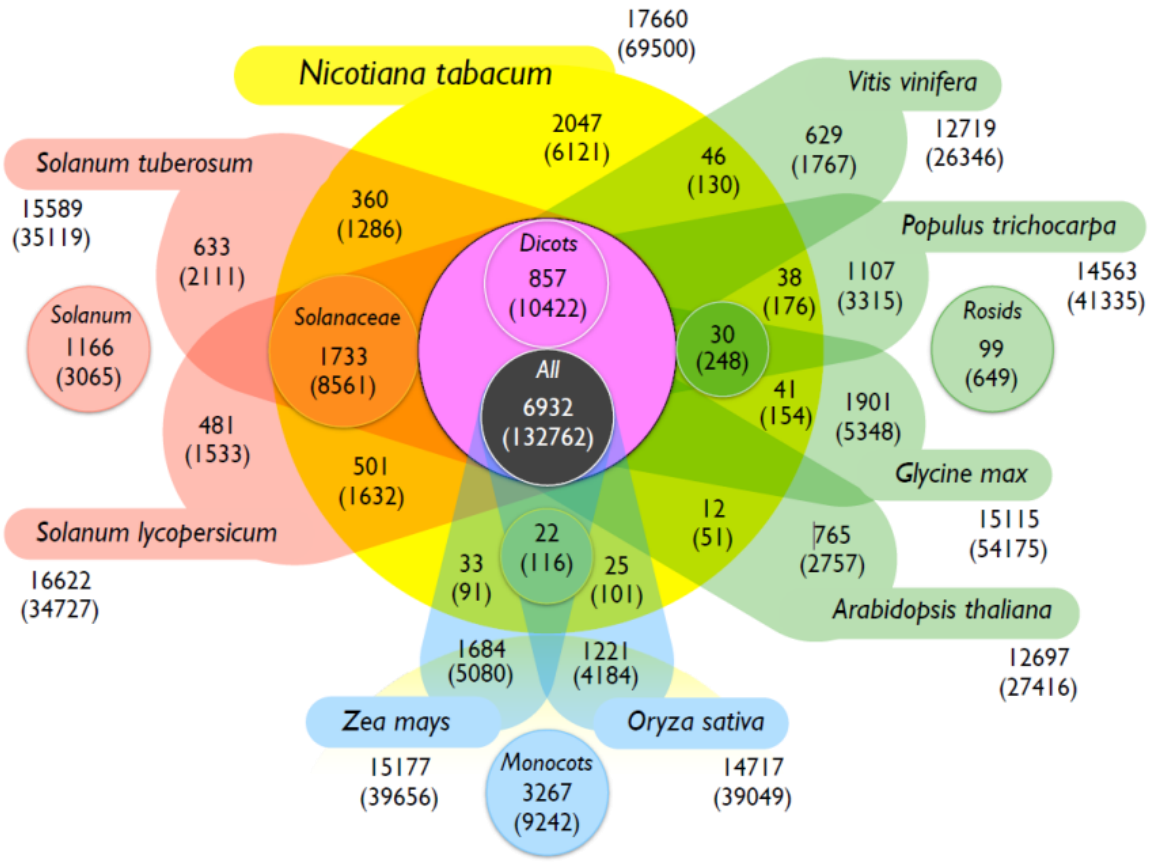

Supplementary Data 3 - Venn diagram of sequenced plant gene families

Venn diagram comparison of gene families between tobacco (yellow), the *Solanum* (potato [*S. tuberosum*] and tomato [*S. lycopersicum*]; red), rosids (grape vine [*Vitis vinifera*], poplar [*Populus trichocarpa*], soybean [*Glycine max*] and *Arabidopsis thaliana*; green) and monocotyledons (rice [*Oryza sativa*] and maize [*Zea mays*]; blue). Number of gene families in each cross over stated, with the total number of genes in these families shown in parentheses.
