# Supplementary material for: A reference genome for Nicotiana tabacum enables map-based cloning of homeologous loci implicated in nitrogen utilization efficiency
Source: BMC Genomics. 2017 Jun 19;18:448. doi: 10.1186/s12864-017-3791-6 (PMC5474855; doi:10.1186/s12864-017-3791-6)
Supplement: Supplementary file 4 — Bar charts and tables summarising functional annotation of tobacco gene models. (PDF 369 kb) [file 12864_2017_3791_MOESM4_ESM.pdf]

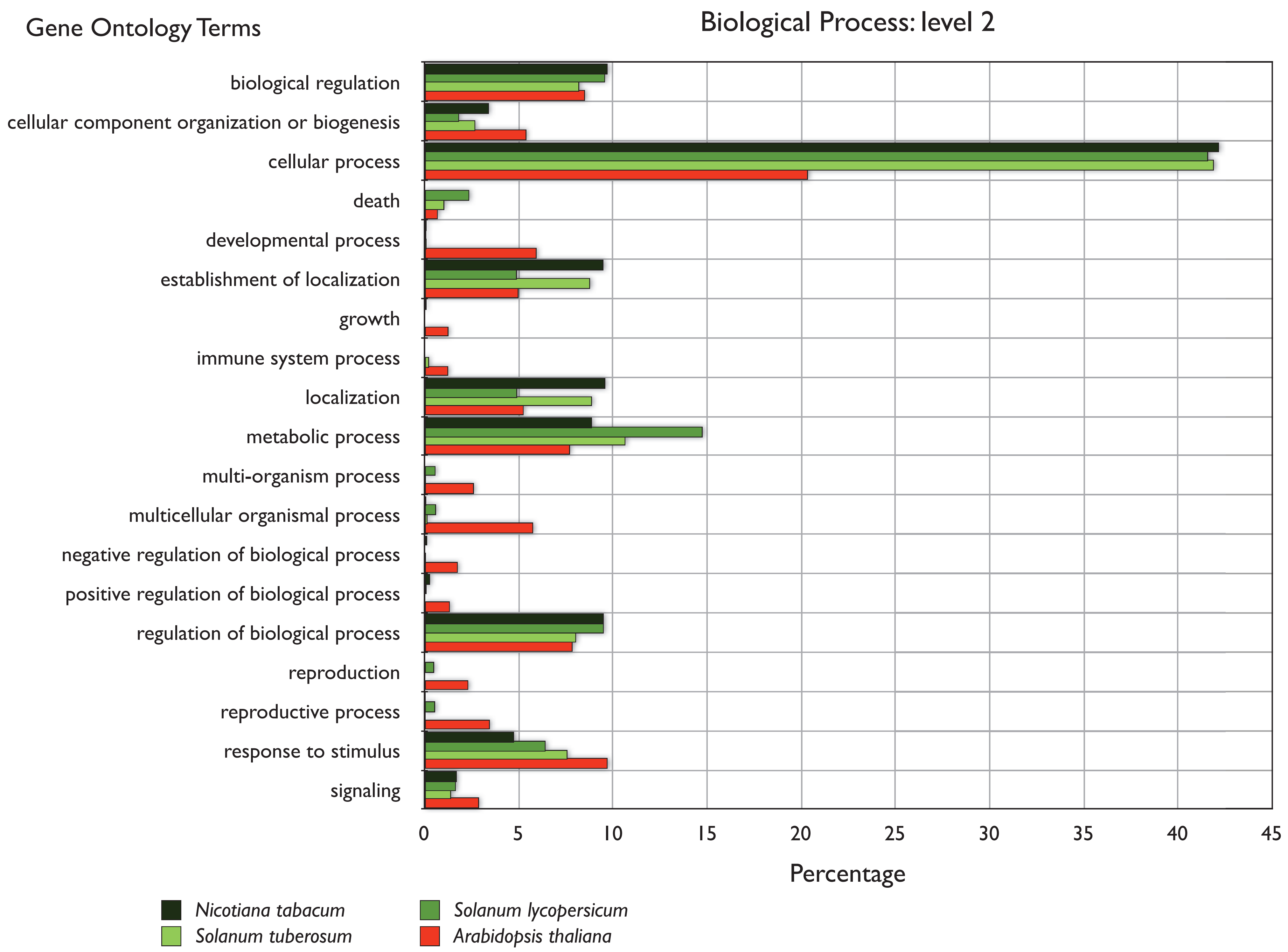

Gene Ontology Terms
Biological Process: level 2
biological regulation
cellular component organization or biogenesis
cellular process
death
developmental process
establishment of localization
growth
immune system process
localization
metabolic process
multi-organism process
multicellular organismal process
negative regulation of biological process
positive regulation of biological process
regulation of biological process
reproduction
reproductive process
response to stimulus
signaling
0
5
10
15
20
25
30
35
40
45
Percentage
Nicotiana tabacum
Solanum lycopersicum
Solanum tuberosum
Arabidopsis thaliana

Gene Ontology Terms Biological Process: level 2

Nicotiana tabacum

GO Terms	#	%
biological regulation	3114	9.71
cellular component organization or biogenesis	1094	3.41
cellular process	13520	42.16
death	3	0.01
developmental process	34	0.11
establishment of localization	3044	9.49
growth	31	0.10
localization	3074	9.59
metabolic process	2850	8.89
multi-organism process	5	0.02
multicellular organismal process	28	0.09
negative regulation of biological process	42	0.13
positive regulation of biological process	92	0.29
regulation of biological process	3045	9.50
reproduction	9	0.03
reproductive process	15	0.05
response to stimulus	1522	4.75
signaling	547	1.71

Solanum tuberosum

GO Terms	#	%
biological regulation	1247	8.20
cell proliferation	10	0.07
cellular component organization or biogenesis	409	2.69
cellular process	6371	41.89
death	158	1.04
developmental process	17	0.11
establishment of localization	1336	8.78
immune system process	35	0.23
localization	1350	8.88
metabolic process	1621	10.66
multi-organism process	9	0.06
multicellular organismal process	23	0.15
negative regulation of biological process	10	0.07
regulation of biological process	1223	8.04
reproductive process	7	0.05
response to stimulus	1153	7.58
signaling	213	1.40

Solanum lycopersicum

GO Terms	#	%
biological regulation	1019	9.58
cellular component organization or biogenesis	195	1.83
cellular process	4422	41.58
death	252	2.37
developmental process		0.08
establishment of localization	520	4.89
localization	522	4.91
metabolic process	1568	14.75
multi-organism process	62	0.58
multicellular organismal process	64	0.60
positive regulation of biological process	11	0.10
regulation of biological process	1011	9.51
reproduction	53	0.50
reproductive process	59	0.55
response to stimulus	683	6.42
signaling	176	1.66

Arabidopsis thaliana

GO Terms	#	%
biological adhesion	97	0.13
biological regulation	6168	8.51
cell proliferation	271	0.37
cellular component organization or biogenesis	3914	5.40
cellular process	14741	20.34
death	504	0.70
developmental process	4303	5.94
establishment of localization	3606	4.98
growth	911	1.26
immune system process	903	1.25
localization	3812	5.26
metabolic process	5588	7.71
multi-organism process	1891	2.61
multicellular organismal process	4175	5.76
negative regulation of biological process	1274	1.76
pigmentation	113	0.16
positive regulation of biological process	970	1.34
regulation of biological process	5690	7.85
reproduction	1681	2.32
reproductive process	2507	3.46
response to stimulus	7041	9.71
rhythmic process	169	0.23
signaling	2090	2.88

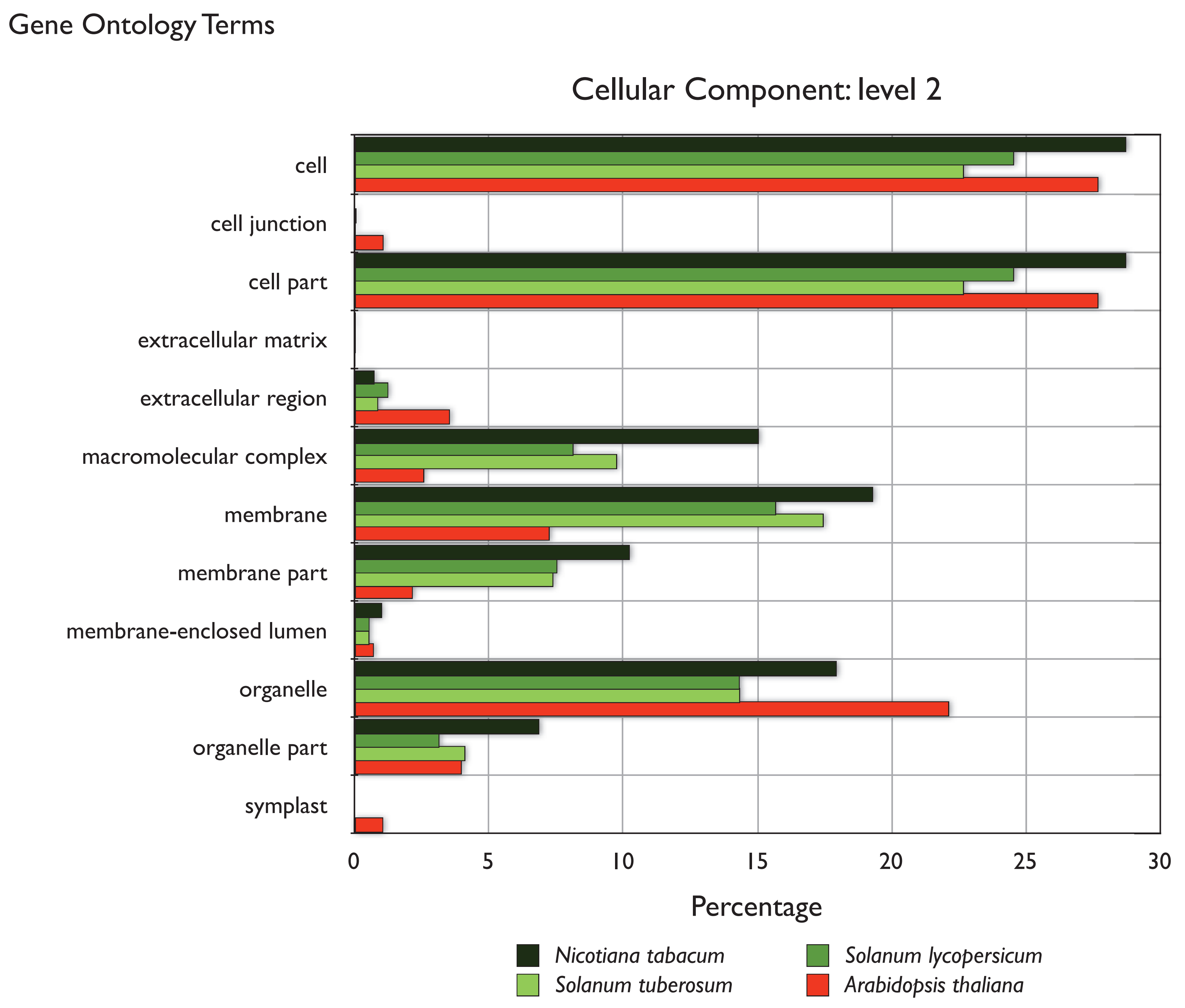
Gene Ontology Terms
Cellular Component: level 2
cell
cell junction
cell part
extracellular matrix
extracellular region
macromolecular complex
membrane
membrane part
membrane-enclosed lumen
organelle
organelle part
symplast
0
5
10
15
20
25
30
Percentage
Nicotiana tabacum
Solanum lycopersicum
Solanum tuberosum
Arabidopsis thaliana

Gene Ontology Terms

Cellular Component: level 2

Nicotiana tabacum

GO Terms	#	%
cell	5550	28.73
cell junction	0	0
cell part	5550	28.73
extracellular matrix	9	0.05
extracellular region	145	0.75
macromolecular complex	2908	15.05
membrane	3731	19.31
membrane part	1981	10.25
membrane-enclosed lumen	199	1.03
organelle	3468	17.95
organelle part	1330	6.88

Solanum lycopersicum

GO Terms	#	%
cell	3634	24.55
cell junction	11	0.07
cell part	3634	24.55
extracellular matrix	6	0.04
extracellular region	186	1.26
extracellular region part	4	0.03
macromolecular complex	1207	8.15
membrane	2323	15.69
membrane part	1118	7.55
membrane-enclosed lumen	83	0.56
organelle	2122	14.34
organelle part	467	3.16
virion	3	0.02
virion part	3	0.02

Solanum tuberosum

GO Terms	#	%
cell	2590	22.68
cell junction	6	0.05
cell part	2590	22.68
extracellular matrix	5	0.04
extracellular region	101	0.88
macromolecular complex	1116	9.77
membrane	1994	17.46
membrane part	845	7.40
membrane-enclosed lumen	63	0.55
organelle	1638	14.35
organelle part	470	4.12

Arabidopsis thaliana

GO Terms	#	%
cell	21882	27.69
cell junction	854	1.08
cell part	21882	27.69
extracellular matrix	22	0.03
extracellular region	2807	3.55
extracellular region part	23	0.03
macromolecular complex	2044	2.59
membrane	5741	7.26
membrane part	1714	2.17
membrane-enclosed lumen	573	0.72
organelle	17491	22.13
organelle part	3152	3.99
symplast	849	1.07

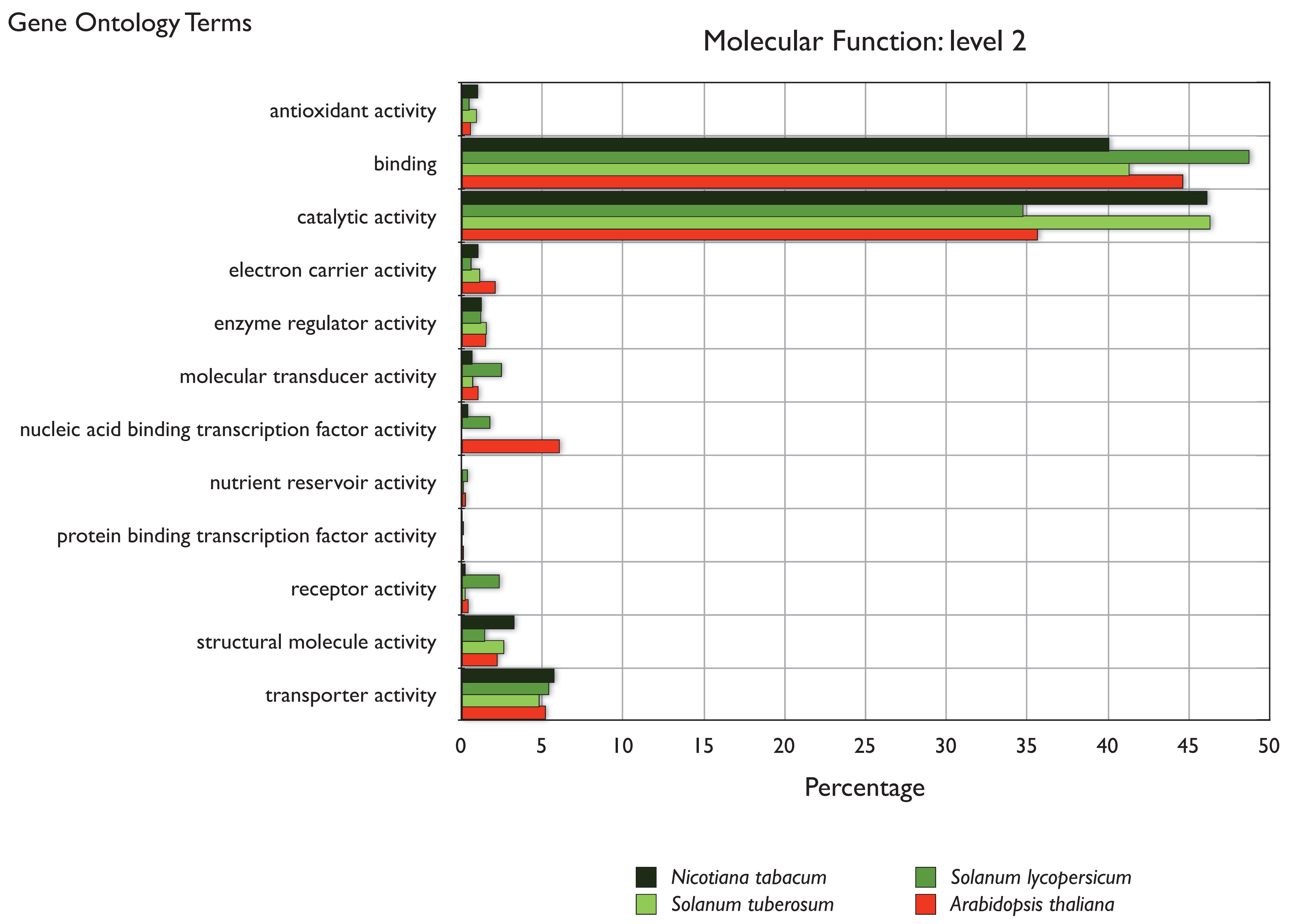
Gene Ontology Terms
Molecular Function: level 2
antioxidant activity
binding
catalytic activity
electron carrier activity
enzyme regulator activity
molecular transducer activity
nucleic acid binding transcription factor activity
nutrient reservoir activity
protein binding transcription factor activity
receptor activity
structural molecule activity
transporter activity
0
5
10
15
20
25
30
35
40
45
50
Percentage
Nicotiana tabacum
Solanum tuberosum
Solanum lycopersicum
Arabidopsis thaliana

Gene Ontology Terms
Molecular Function: level 2

Nicotiana tabacum

GO Terms	#	%
antioxidant activity	330	1.03
binding	12874	40.07
catalytic activity	14826	46.14
electron carrier activity	335	1.04
enzyme regulator activity	404	1.26
molecular transducer activity	220	0.68
nucleic acid binding transcription factor activity	134	0.42
protein binding transcription factor activity	24	0.07
receptor activity	79	0.25
structural molecule activity	1055	3.28
transporter activity	1849	5.75

Solanum lycopersicum

GO Terms	#	%
antioxidant activity	87	0.49
binding	8620	48.74
catalytic activity	6148	34.76
electron carrier activity	108	0.61
enzyme regulator activity	215	1.22
metallochaperone activity	10	0.06
molecular transducer activity	440	2.49
nucleic acid binding transcription factor activity	315	1.78
nutrient reservoir activity	69	0.39
protein binding transcription factor activity	24	0.14
receptor activity	418	2.36
structural molecule activity	258	1.46
transporter activity	958	5.42

Solanum tuberosum

GO Terms	#	%
antioxidant activity	170	0.95
binding	7362	41.32
catalytic activity	8255	46.33
electron carrier activity	205	1.15
enzyme regulator activity	278	1.56
molecular transducer activity	126	0.71
nucleic acid binding transcription factor activity	9	0.05
nutrient reservoir activity	27	0.15
protein binding transcription factor activity	11	0.06
receptor activity	45	0.25
structural molecule activity	470	2.64
transporter activity	859	4.82

Arabidopsis thaliana

GO Terms	#	%
antioxidant activity	143	0.58
binding	10998	44.64
catalytic activity	8783	35.65
electron carrier activity	519	2.11
enzyme regulator activity	375	1.52
molecular transducer activity	259	1.05
nucleic acid binding transcription factor activity	1497	6.08
nutrient reservoir activity	65	0.26
protein binding transcription factor activity	35	0.14
receptor activity	108	0.44
structural molecule activity	549	2.23
transporter activity	1283	5.21

The 50 Most common
InterPro Domains

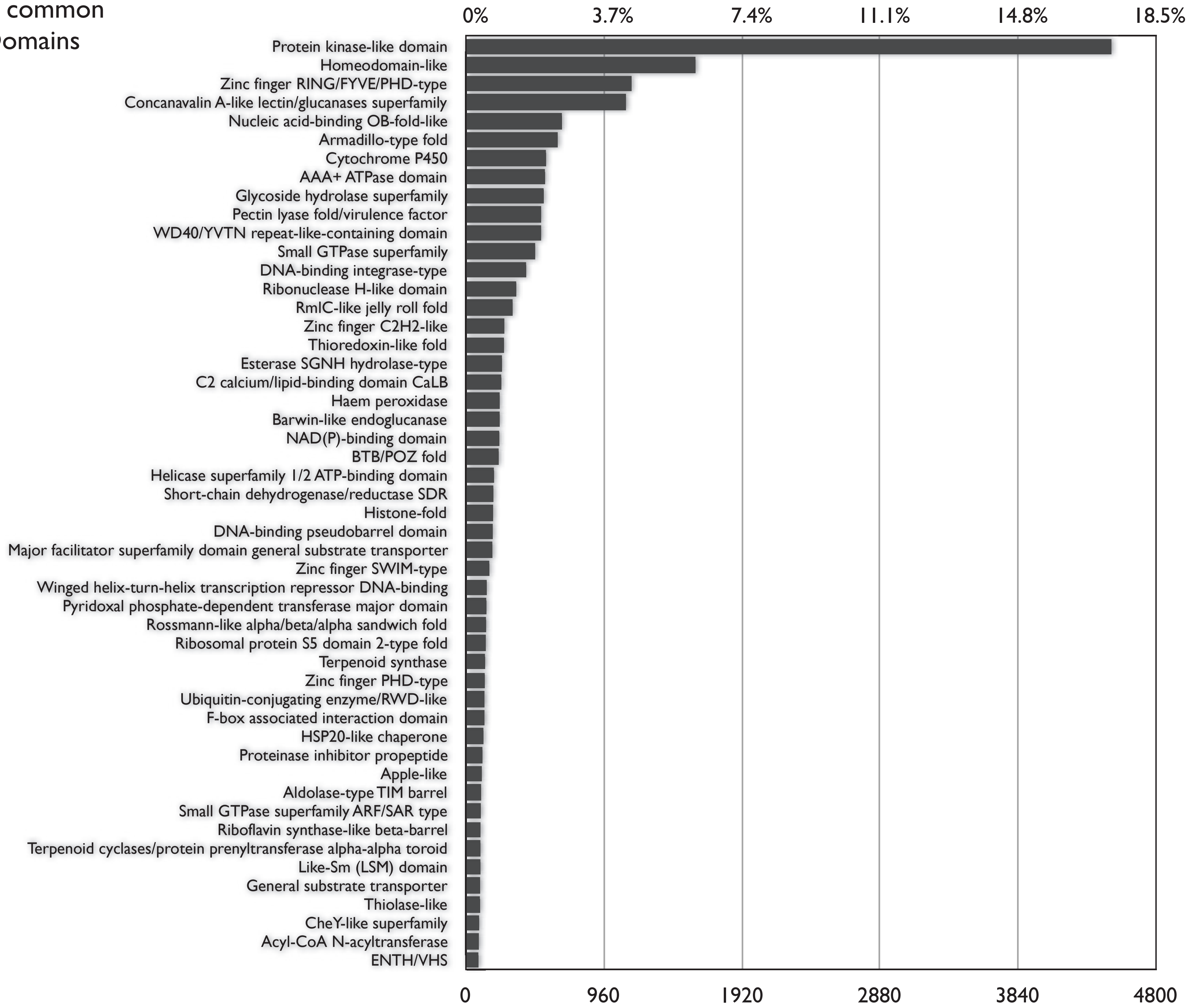

The 50 Most common InterPro Domains

InterPro_term	gene_num	Percentage	Description
IPR011009	4489	17.325	Protein kinase-like domain
IPR009057	1596	6.160	Homeodomain-like
IPR013083	1152	4.446	Zinc finger, RING/FYVE/PHD-type
IPR008985	1113	4.295	Concanavalin A-like lectin/glucanases superfamily
IPR016027	667	2.574	Nucleic acid-binding, OB-fold-like
IPR016024	638	2.462	Armadillo-type fold
IPR001128	556	2.146	Cytochrome P450
IPR003593	549	2.119	AAA+ ATPase domain
IPR017853	540	2.084	Glycoside hydrolase, superfamily
IPR011050	523	2.018	Pectin lyase fold/virulence factor
IPR015943	523	2.018	WD40/YVTN repeat-like-containing domain
IPR001806	481	1.856	Small GTPase superfamily
IPR016177	418	1.613	DNA-binding, integrase-type
IPR012337	349	1.347	Ribonuclease H-like domain
IPR014710	324	1.250	RmlC-like jelly roll fold
IPR015880	267	1.030	Zinc finger, C2H2-like
IPR012336	264	1.019	Thioredoxin-like fold
IPR013830	250	0.965	Esterase, SGNH hydrolase-type
IPR008973	245	0.946	C2 calcium/lipid-binding domain, CaLB
IPR010255	234	0.903	Haem peroxidase
IPR014733	234	0.903	Barwin-like endoglucanase
IPR016040	231	0.892	NAD(P)-binding domain
IPR011333	227	0.876	BTB/POZ fold
IPR014001	194	0.749	Helicase, superfamily 1/2, ATP-binding domain
IPR002198	190	0.733	Short-chain dehydrogenase/reductase SDR
IPR009072	189	0.729	Histone-fold
IPR015300	186	0.718	DNA-binding pseudobarrel domain
IPR016196	183	0.706	Major facilitator superfamily domain, general substrate transporter
IPR007527	162	0.625	Zinc finger, SWIM-type

InterPro_term	gene_num	Percentage	Description
IPR011991	144	0.556	Winged helix-turn-helix transcription repressor DNA-binding
IPR015424	141	0.544	Pyridoxal phosphate-dependent transferase, major domain
IPR014729	139	0.536	Rossmann-like alpha/beta/alpha sandwich fold
IPR020568	138	0.533	Ribosomal protein S5 domain 2-type fold
IPR008949	132	0.509	Terpenoid synthase
IPR001965	130	0.502	Zinc finger, PHD-type
IPR016135	129	0.498	Ubiquitin-conjugating enzyme/RWD-like
IPR017451	129	0.498	F-box associated interaction domain
IPR008978	121	0.467	HSP20-like chaperone
IPR009020	114	0.440	Proteinase inhibitor, propeptide
IPR003609	110	0.425	Apple-like
IPR013785	105	0.405	Aldolase-type TIM barrel
IPR006689	103	0.398	Small GTPase superfamily, ARF/SAR type
IPR017938	101	0.390	Riboflavin synthase-like beta-barrel
IPR008930	100	0.386	Terpenoid cyclases/protein prenyltransferase alpha-alpha toroid
IPR010920	100	0.386	Like-Sm (LSM) domain
IPR005828	98	0.378	General substrate transporter
IPR016039	97	0.374	Thiolase-like
IPR011006	91	0.351	CheY-like superfamily
IPR016181	90	0.347	Acyl-CoA N-acyltransferase
IPR008942	86	0.332	ENTH/VHS

2169 Protein Kinases were found by HMM

Class 1 - Transmembrane Receptor Kinase and Related non-Transmembrane Kinases

**Group 1.1**		
PPC:1.1.1	Leucine-rich transmembrane protein kinase/Strubbelig Receptor Family 1	17
PPC:1.1.2	Putative protein kinase/Ser_thr kinase like protein/Putative receptor-like protein kinase	4
PPC:1.1.3	Putative protein kinase/Putative receptor-like protein kinase	20
**Group 1.2 - Receptor Like Cytoplasmic Kinase**		
PPC:1.2.1	Receptor Like Cytoplasmic Kinase VIII	22
PPC:1.2.2	Receptor Like Cytoplasmic Kinase VII	114
PPC:1.2.5	ser_thr protein kinase like protein	1
**Group 1.3**		
PPC:1.3.1	Receptor-like protein kinase	38
PPC:1.3.2	Leucine Rich Repeat Receptor VIII	20
PPC:1.3.3	Leucine Rich Repeat Receptor Kinase I & Unknown Receptor Kinase I	8
PPC:1.3.7	Receptor like protein kinase/Receptor lectin kinase like protein	4
**Group 1.4 - Crinkly 4_Like Kinase**		
PPC:1.4.1	Crinkly 4 Like Kinase	23
PPC:1.4.2	Tousled like kinase	1
**Group 1.5**		
PPC:1.5.1	Wall Associated Kinase-like Kinase	42
PPC:1.5.2	LRK10 Like Kinase (Type 1)	17
PPC:1.5.3	Receptor Like Cytoplasmic Kinase IV	4
**Group 1.6**		
PPC:1.6.1	Leucine Rich Repeat Kinase IX	22
PPC:1.6.2	Plant External Response Like Kinase	45
PPC:1.6.3	Receptor Like Cytoplasmic Kinase V	15
PPC:1.6.4	RKF3 Like Kinase	5
**Group 1.7 - S-Domain and Duf26 Domain Kinase**		
PPC:1.7.1	S Domain Kinase (Type 1)	26
PPC:1.7.2	Domain of Unknown Function 26 (DUF26) Kinase	147
**Group 1.8 - Leucine Rich Repeat Receptor Kinase**		
PPC:1.8.1	Leucine Rich Repeat Kinase I	12

**Group 1.9**		
PPC:1.9.1	CRPK1 Like Kinase (Types 1 and 2)	43
PPC:1.9.2	S Domain Kinase (Type 2)	108
PPC:1.9.3	Putative receptor like protein kinase	8
PPC:1.9.5	Putative LRR receptor-like protein kinase/Receptor protein kinase like protein	6
**Group 1.10 - Receptor Like Cytoplasmic Kinase**		
PPC:1.10.1	Receptor Like Cytoplasmic Kinase VI	37
**Group 1.11 - Legume Lectin Domain Kinase**		
PPC:1.11.1	Legume Lectin Domain Kinase	49
**Group 1.12 - Leucine Rich Repeat Receptor Kinase**		
PPC:1.12.1	Leucine Rich Repeat Kinase X	12
PPC:1.12.2	Leucine Rich Repeat Kinase II & X	36
PPC:1.12.3	Leucine Rich Repeat Kinase VII	18
PPC:1.12.4	Leucine Rich Repeat Kinase XI & XII	238
PPC:1.12.5	Leucine Rich Repeat Kinase X	6
**Group 1.13 - Leucine Rich Repeat Receptor Kinase**		
PPC:1.13.1	Leucine Rich Repeat Kinase VII	2
PPC:1.13.2	Leucine Rich Repeat Kinase IV	6
PPC:1.13.3	Leucine Rich Repeat Kinase III	42
PPC:1.13.4	Leucine Rich Repeat Kinase III	14
PPC:1.13.5	Leucine Rich Repeat Kinase III	7
**Group 1.14 -**		
PPC:1.14.1	Crinkly 4 Like Kinase	7
PPC:1.14.2	Receptor Like Cytoplasmic Kinase IX	28
**Group 1.15 -**		
PPC:1.15.1	Receptor Like Cytoplasmic Kinase I	9
PPC:1.15.2	Leucine Rich Repeat Kinase VI	6
PPC:1.15.3	Leucine Rich Repeat Kinase IV	4
**Group 1.16 - Receptor Like Cytoplasmic Kinase**		
PPC:1.16.1	Receptor Like Cytoplasmic Kinase II	18
**Group 1.17 - Wall Associated Kinase**		
PPC:1.17.1	Wall Associated Kinase	1
**Group 1.Other**		
PPC:1.Other	Other Protein Kinase	26

2169 Protein Kinases were found by HMM

Class 2 - ATN1/CTR1/EDR1/GmPK6 like Kinase

**Group 2.1 - ATN1/CTR1/EDR1/GmPK6 like Kinase**		
PPC:2.1.1	Light Sensor Kinase	6
PPC:2.1.2	Ankyrin Repeat Domain Kinase	15
PPC:2.1.3	CTR1/EDR1 Kinase	34
PPC:2.1.4	GmPK6/AtMRK1 Family	53
PPC:2.1.5	ATN1 Like Family	9
PPC:2.1.6	Unknown Function Kinase	3
**Group 2.2 - Unknown Function Protein Kinase**		
PPC:2.2.1	Unknown Function Kinase	4
PPC:2.2.2	C-terminal Ankyrin Repeat Domain Kinase	2

Class 3 - Casein Kinase I

**Group 3.1 - Casein Kinase I**		
PPC:3.1.1	Casein Kinase I Family	38

Class 5 - Other and Unclassified Protein Kinase

**Group 5.1 - Other Protein Kinase**		
PPC:5.1.1	Other Kinase	7
PPC:5.1.2	Other Kinase	4
**Group 5.2 - Other Protein Kinase**		
PPC:5.2.1	Other Kinase	2

Class 4 - Non-Transmembrane Protein Kinases

**Group 4.1 - Mitogen Activated Protein Kinase Kinase Kinase (MAP3K)**		
PPC:4.1.1	MAP3K	29
PPC:4.1.2	STE20-PAK Like Protein Kinase	22
PPC:4.1.3	MAP2K	5
PPC:4.1.4	MAP2K	8
PPC:4.1.5	Possible MAP2K	3
PPC:4.1.5.1	WNK like kinase - with no lysine kinase	26
PPC:4.1.6	Unknown Function Kinase	15
PPC:4.1.7	APG1 Like Kinase	8
**Group 4.2 - Calcium Response Kinase**		
PPC:4.2.1	Calcium Dependent Protein Kinase	81
PPC:4.2.2	Phosphoenolpyruvate Carboxylase Kinase	9
PPC:4.2.3	Calcium/Calmodulin Dependent Protein Kinase (CCamK)	2
PPC:4.2.4	SNF1 Related Protein Kinase (SnRK)	111
PPC:4.2.5	Unknown Function Kinase	12
PPC:4.2.6	IRE/NPH/PI dependent/S6 Kinase	73
PPC:4.2.7	ELM1/PAK1/TOS3 Like Kinase	4
**Group 4.3 - Unknown Function Protein Kinase**		
PPC:4.3.1	Unknown Function Kinase	2
PPC:4.3.2	Unknown Function Kinase	3
**Group 4.4 - Unknown Function Protein Kinase**		
PPC:4.4.1	Unknown Function Kinase	69
PPC:4.4.2	Unknown Function Kinase	2
PPC:4.4.3	Unknown Function Kinase	3
**Group 4.5 - MAPK/CDC/CK2/GSK Kinases**		
PPC:4.5.1	MAPK Family	42
PPC:4.5.1.1	Male grem cell-associated kinase (mak)	9
PPC:4.5.2	CDC2 Like Kinase Family	60
PPC:4.5.3	Casein Kinase II Family	6
PPC:4.5.4	GSK3/Shaggy Like Protein Kinase Family	23
PPC:4.5.5	Unknown Function Kinase	2
PPC:4.5.6	LAMMER Kinase Family	8
PPC:4.5.7	Unknown Function Kinase	9
PPC:4.5.8	Unknown Function Kinase	8

2169 Protein Kinases were found by HMM (sorted by ocurrence)

PK	#
Leucine Rich Repeat Kinase XI & XII	238
Domain of Unknown Function 26 (DUF26) Kinase	147
Unknown Function Kinase	132
Receptor Like Cytoplasmic Kinase VII	114
SNF1 Related Protein Kinase (SnRK)	111
S Domain Kinase (Type 2)	108
Calcium Dependent Protein Kinase	81
IRE/NPH/PI dependent/S6 Kinase	73
Leucine Rich Repeat Kinase III	63
CDC2 Like Kinase Family	60
GmPK6/AtMRK1 Family	53
Legume Lectin Domain Kinase	49
Plant External Response Like Kinase	45
CRPK1 Like Kinase (Types 1 and 2)	43
MAPK Family	42
Wall Associated Kinase-like Kinase	42
Casein Kinase I Family	38
Receptor-like protein kinase	38
Receptor Like Cytoplasmic Kinase VI	37
Leucine Rich Repeat Kinase II & X	36
CTR1/EDR1 Kinase	34
Crinkly 4 Like Kinase	30
MAP3K	29
Receptor Like Cytoplasmic Kinase IX	28
S Domain Kinase (Type 1)	26
WNK like kinase - with no lysine kinase	26
Other Protein Kinase	25
GSK3/Shaggy Like Protein Kinase Family	23
Leucine Rich Repeat Kinase IX	22
Receptor Like Cytoplasmic Kinase VIII	22
STE20-PAK Like Protein Kinase	22
Leucine Rich Repeat Kinase VII	20
Leucine Rich Repeat Receptor VIII	20
Putative protein kinase/Putative receptor-like protein kinase	20

PK	#
Leucine Rich Repeat Kinase X	18
Receptor Like Cytoplasmic Kinase II	18
LRK10 Like Kinase (Type 1)	17
Leucine-rich transmembrane protein kinase/Strubbelig Receptor Family 1	17
Ankyrin Repeat Domain Kinase	15
Receptor Like Cytoplasmic Kinase V	15
MAP2K	13
Other Kinase	13
Leucine Rich Repeat Kinase I	12
Leucine Rich Repeat Kinase IV	10
ATN1 Like Family	9
Male grem cell-associated kinase (mak)	9
Phosphoenolpyruvate Carboxylase Kinase	9
Receptor Like Cytoplasmic Kinase I	9
APG1 Like Kinase	8
LAMMER Kinase Family	8
Leucine Rich Repeat Receptor Kinase I & Unknown Receptor Kinase I	8
Putative receptor like protein kinase	8
Casein Kinase II Family	6
Leucine Rich Repeat Kinase VI	6
Light Sensor Kinase	6
Putative LRR receptor-like protein kinase/Receptor protein kinase like protein	6
RKF3 Like Kinase	5
ELM1/PAK1/TOS3 Like Kinase	4
Putative protein kinase/Ser_thr kinase like protein/Putative receptor-like protein kinase	4
Receptor Like Cytoplasmic Kinase IV	4
Receptor like protein kinase/Receptor lectin kinase like protein	4
Possible MAP2K	3
C-terminal Ankyrin Repeat Domain Kinase	2
Calcium/Calmodulin Dependent Protein Kinase (CCamK)	2
Tousled like kinase	1
Wall Associated Kinase	1
ser_thr protein kinase like protein	1

4713 Transcription factor were found by HMM (sorted by ocurrence)

TF	#
MYB	406
AP2-EREBP	374
NAC	263
bHLH	247
MADS	219
C2H2	215
HB	185
WRKY	151
C3H	141
bZIP	136
Orphans	133
ABI3VP1	132
FAR1	117
LOB	106
GRAS	102
CCAAT	92
PHD	81
G2-like	80
mTERF	80
GNAT	79

TF	#
SET	77
SNF2	74
C2C2-Dof	72
TCP	62
AUX/IAA	57
C2C2-GATA	57
Trihelix	56
HSF	53
SBP	51
ARF	45
SWI/SNF-BAF60b	42
OFP	40
Jumonji	36
TRAF	34
zf-HD	33
RWP-RK	31
FHA	29
TUB	26
Tify	26
GRF	24

TF	#
HMG	23
BES1	22
PLATZ	22
ARR-B	20
Alfin-like	20
GeBP	20
C2C2-CO-like	19
ARID	18
SRS	18
C2C2-YABBY	17
IWS1	17
LIM	17
BSD	16
EIL	16
DDT	14
Sigma70-like	14
TAZ	14
E2F-DP	13
BBR/BPC	12
CAMTA	11

TF	#
SWI/SNF-SWI3	10
LUG	9
Rcd1-like	9
CPP	8
Pseudo ARR-B	8
Coactivator p15	7
DBP	7
MBF1	7
CSD	6
S1Fa-like	6
PBF-2-like	5
ULT	5
VOZ	4
HRT	3
SAP	3
LFY	2
MED6	2
MED7	2
SOH1	2
NOZZLE	1

4713 Transcription factor were found by HMM (alphabetically sorted)

3972 Transcription factors

TF	#
ABI3VP1	132
Alfin-like	20
AP2-EREBP	374
ARF	45
ARR-B	20
BBR/BPC	12
BES1	22
bHLH	247
BSD	16
bZIP	136
C2C2-CO-like	19
C2C2-Dof	72
C2C2-GATA	57
C2C2-YABBY	17
C2H2	215

TF	#
C3H	141
CAMTA	11
CCAAT	92
CPP	8
CSD	6
DBP	7
E2F-DP	13
EIL	16
FAR1	117
FHA	29
G2-like	80
GeBP	20
GRAS	102
GRF	24
HB	185

TF	#
HRT	3
HSF	53
LFY	2
LIM	17
LOB	106
MADS	219
mTERF	80
MYB	406
NAC	263
NOZZLE	1
OFP	40
PBF-2-like	5
PLATZ	22
RWP-RK	31
S1Fa-like	6

TF	#
SAP	3
SBP	51
Sigma70-like	14
SRS	18
TAZ	14
TCP	62
Tify	26
Trihelix	56
TUB	26
ULT	5
VOZ	4
WRKY	151
zf-HD	33

4713 Transcription factor were found by HMM (alphabetically sorted)

741 Transcriptional regulators

**ARID**	18
**AUX/IAA**	57
**Coactivator p15**	7
**DDT**	14
**GNAT**	79
**HMG**	23
**IWS1**	17
**Jumonji**	36
**LUG**	9
**MED6**	2
**MED7**	2
**MBF1**	7
**Orphans**	133
**PHD**	81
**Pseudo ARR-B**	8
**Rcd1-like**	9
**SET**	77
**SNF2**	74
**SOH1**	2
**SWI/SNF-BAF60b**	42
**SWI/SNF-SWI3**	10
**TRAF**	34
