# Supplementary material for: A reference genome for Nicotiana tabacum enables map-based cloning of homeologous loci implicated in nitrogen utilization efficiency
Source: BMC Genomics. 2017 Jun 19;18:448. doi: 10.1186/s12864-017-3791-6 (PMC5474855; doi:10.1186/s12864-017-3791-6)
Supplement: Supplementary file 5 — Venn diagram showing numbers of tobacco gene models scored as expressed in root, shoot, and shoot apex samples. (PDF 420 kb) [file 12864_2017_3791_MOESM5_ESM.pdf]

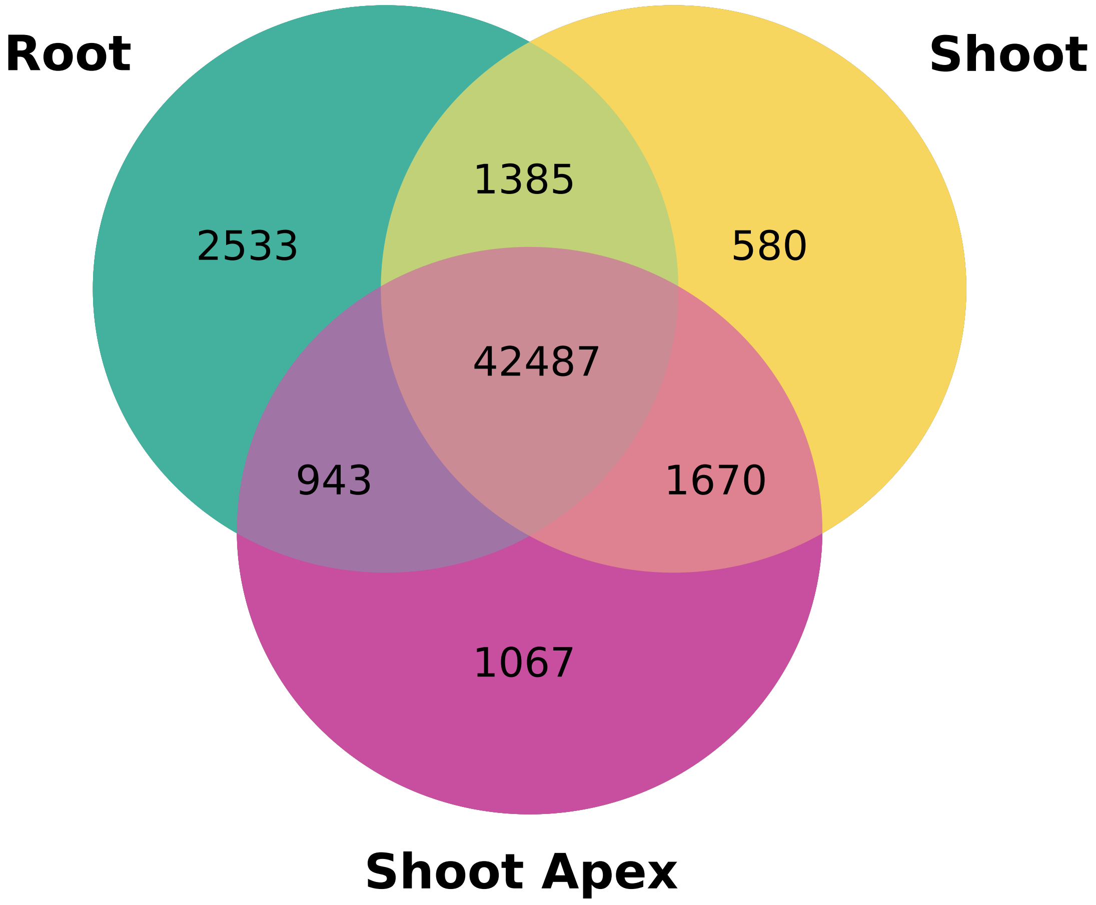

Sample Name	Tissue	Time Point	Replicate	Raw Reads	Filtered Reads	% Filtered	Reads Mapped	% Mapped	% Duplication
A6140	Root	ZT0	R1	43870402	43273979	98.64	17070456	39.45	41.8
A6141	Root	ZT0	R2	32377489	31991359	98.81	11904484	37.21	45.44
A6142	Root	ZT0	R3	40835020	40437551	99.03	15328554	37.91	44.37
A6149	Shoot	ZT0	R1	32327637	32093901	99.28	12418252	38.69	38.65
A6150	Shoot	ZT0	R2	40623084	39815155	98.01	15699541	39.43	39.82
A6151	Shoot	ZT0	R3	35337160	34999827	99.05	12759085	36.45	42.64
A6158	ShootApex	ZT0	R1	28165222	28018049	99.48	11384982	40.63	38.72
A6159	ShootApex	ZT0	R2	37969866	37062211	97.61	13673051	36.89	48.05
A6160	ShootApex	ZT0	R3	33266812	33009200	99.23	13606778	41.22	44.65
A6167	Root	ZT6	R1	39635426	38805901	97.91	5273883	13.59	74.64
A6168	Root	ZT6	R2	34278254	33776361	98.54	8036587	23.79	59.75
A6169	Root	ZT6	R3	33479877	33227956	99.25	13146825	39.57	42.04
A6176	Shoot	ZT6	R1	50166956	48156700	95.99	5458175	11.33	89.96
A6177	Shoot	ZT6	R2	53042894	52672970	99.30	21311841	40.46	42.23
A6178	Shoot	ZT6	R3	30430562	30230218	99.34	12031531	39.80	41.83
A6185	ShootApex	ZT6	R1	54461029	53989810	99.13	21537148	39.89	48.1
A6186	ShootApex	ZT6	R2	41107412	40852094	99.38	17525739	42.90	43.33
A6187	ShootApex	ZT6	R3	36453862	36219573	99.36	14675978	40.52	43.73
A6194	Root	ZT12	R1	54140362	53760731	99.30	21360630	39.73	41.69
A6195	Root	ZT12	R2	40354648	39967798	99.04	15612819	39.06	36.1
A6196	Root	ZT12	R3	30431742	30215084	99.29	12054581	39.90	41.67
A6203	Shoot	ZT12	R1	36055980	35790086	99.26	13673743	38.21	41.29
A6204	Shoot	ZT12	R2	38943340	38582281	99.07	14485476	37.54	36.57
A6205	Shoot	ZT12	R3	32873932	32642330	99.30	13036886	39.94	34.31
A6212	ShootApex	ZT12	R1	56610222	55350519	97.77	21162372	38.23	27.45
A6213	ShootApex	ZT12	R2	39566661	39273575	99.26	15837289	40.33	42.8
A6214	ShootApex	ZT12	R3	57139987	56753817	99.32	22763443	40.11	47.35
A6221	Root	ZT18	R1	34302320	33880198	98.77	12024593	35.49	41.25
A6222	Root	ZT18	R2	38093675	24268245	63.71	855912	3.53	39.77
A6223	Root	ZT18	R3	40797351	40494453	99.26	16284365	40.21	40.21
A6230	Shoot	ZT18	R1	31954364	31593165	98.87	12535413	39.68	34.9
A6231	Shoot	ZT18	R2	43330745	41524157	95.83	4835524	11.65	80.88
A6232	Shoot	ZT18	R3	32557546	32261239	99.09	12686586	39.32	37.41
A6239	ShootApex	ZT18	R1	31713996	31574094	99.56	12946068	41.00	36.55
A6240	ShootApex	ZT18	R2	29910041	29763220	99.51	12052232	40.49	39.42
A6241	ShootApex	ZT18	R3	41946226	41534139	99.02	16046566	38.63	40.01
