# Supplementary material for: A reference genome for Nicotiana tabacum enables map-based cloning of homeologous loci implicated in nitrogen utilization efficiency
Source: BMC Genomics. 2017 Jun 19;18:448. doi: 10.1186/s12864-017-3791-6 (PMC5474855; doi:10.1186/s12864-017-3791-6)
Supplement: Supplementary file 6 — Alignment of cDNA sequence for YB1 (NtEGY2) and YB2 (NtEGY1) alleles from K326 and TN90 cultivars. (PDF 183 kb) [file 12864_2017_3791_MOESM6_ESM.pdf]

```
Yb1_cDNA_K326    1 ATGGGAACGCTAACGAGCTGCAGTTTCAGCTCAATGAATATAAGGTTCCGTTTGAATCCTCCAGTTAATTACACTTTCAG
Yb1_cDNA_TN90    1 ATGGGAACGCTAACGAGCTGCAGTTTCAGCACAATGAATATAAGGTTCCGTTTGAATCCTCCAGTTAATTACACTTTCAG
Yb2_cDNA_K326    1 ATGGGAACGCTAACGAGCTGCAGTTTCAGCACAATGAATATAAGGTTCCGTTTGAATCCTCCAGTTAATCACAGTTTCAG
Yb2_cDNA_TN90    1 ATGGGAACGCTAACGAGCTGCAGTTTCAGCACAATGAATATAAGGTTCCGTTTGAATCCTCCAGTTAATCACAGTTTCAG

Yb1_cDNA_K326   81 TCGAAGAATCCAATTGAAGAGAATGTCCAAACGGAATTTCGGTCGATTGATTATTAGGTGTAGTAGCGGTAGTAGTGGCA
Yb1_cDNA_TN90   81 TCGAAGAATCCAATTGAAGAGAATGTCCAAACGGAATTTCGGTCGATTGATTATTAGGTGTAGTAGCGGTAGTAGTGGCA
Yb2_cDNA_K326   81 TCGAAGAATCCAATTGAAGAGAATGTCCAAACGGAATTTCGGTAGATTGATTATTAGGTGTAGT---------AGTGGAA
Yb2_cDNA_TN90   81 TCGAAGAATCCAATTGAAGAGAATGTCCAAACGGAATTTCGGTAGATTGATTATTAGGTGTAGT---------AGTGGAA

Yb1_cDNA_K326  161 ATGGCAGTAGCAATGACAGTGGTAGCAGTAGCGATGGGAAATTGGAAAAGGATTCTTCAAATTTGGCTACAGTTACTGAA
Yb1_cDNA_TN90  161 ATGGCAGTAGCAATGACAGTGGTAGCAGTAGCGATGGGAAATTGGAAAAGGATTCTTCAAATTTGGCTACAGTTACTGAA
Yb2_cDNA_K326  152 ATGGCAGTAGCAATAACAATGGCAGCAGTAGCGATGGGAAATTGGAAAAGGATTCTTCAAATTTAGCTACAGTTACTGAA
Yb2_cDNA_TN90  152 ATGGCAGTAGCAATAACAATGGCAGCAGTAGCGATGGGAAATTGGAAAAGGATTCTTCAAATTTAGCTACAGTTACTGAA

Yb1_cDNA_K326  241 GAAACCACTGAAGAAAGGAACGGCGGCGGTGGCGCCAGCGGTGTGGAAAATGATTCGGATGATTCTCCGGTGTCAATTTC
Yb1_cDNA_TN90  241 GAAACCACTGAAGAAAGGAACGGCGGCGGTGGCGCCAGCGGTGTGGAAAATGATTCGGATGATTCTCCGGTGTCAATTTC
Yb2_cDNA_K326  232 GAAACCACTGAAGAAAGGAACGGCGGCGGTGGCGCCAGCGGTGTGGAAAATGATTCCGAGGATTCTCCGGTGTCAATTTC
Yb2_cDNA_TN90  232 GAAACCACTGAAGAAAGGAACGGCGGCGGTGGCGCCAGCGGTGTGGAAAATGATTCCGAGGATTCTCCGGTGTCAATTTC

Yb1_cDNA_K326  321 TTCCAGACCAACAATATCCACTGTTGGATCAACTTATAATAATTTCCAAGTAGATTCTTTTAAGTTGATGGAACTTCTTG
Yb1_cDNA_TN90  321 TTCCAGACCAACAATATCCACTGTTGGATCAACTTATAATAATTTCCAAGTAGATTCTTTTAAGTTGATGGAACTTCTTG
Yb2_cDNA_K326  312 TTCCAGACCAACAATATCCACGGTTGGATCAACTTATAATAATTTCCAAGTAGATTCTTTTAAGTTGATGGAACTTCTTG
Yb2_cDNA_TN90  312 TTCCAGACCAACAATATCCACGGTTGGATCAACTTATAATAATTTCCAAGTAGATTCTTTTAAGTTGATGGAACTTCTTG

Yb1_cDNA_K326  401 GACCAGAAAAGGTTGATCCCAGTGATGTGAAGTTCATTAAGGAAAAGTTATTTGGCTACTCTACTTTTTGGGTGACTAAA
Yb1_cDNA_TN90  401 GACCAGAAAAGGTTGATCCCAGTGATGTGAAGTTCATTAAGGAAAAGTTATTTGGCTACTCTACTTTTTGGGTGACTAAA
Yb2_cDNA_K326  392 GACCAGAAAAGGTTGATCCCAGTGATGTGAAGATAATTAAGGAAAAGTTATTTGGCTACTCTACTTTTTGGGTGACTAAA
Yb2_cDNA_TN90  392 GACCAGAAAAGGTTGATCCCAGTGATGTGAAGATAATTAAGGAAAAGTTATTTGGCTACTCTACTTTTTGGGTGACTAAA

Yb1_cDNA_K326  481 GAAGAACCATTTGGAGATCTTGGAGAGGGCATTCTTTTCCTTGGGAATCTTAGAGGAAAGAGGGAGGATGTTTTTGCCAA
Yb1_cDNA_TN90  481 GAAGAACCATTTGGAGATCTTGGAGAGGGCATTCTTTTCCTTGGGAATCTTAGAGGAAAGAGGGAGGATGTTTTTGCCAA
Yb2_cDNA_K326  472 GAAGAACCATTTGGAGATCTTGGAGAGGGCATTCTTTTCCTTGGGAACCTTAGAGGAAAGAGGGAGGATGTTTTTGCCAA
Yb2_cDNA_TN90  472 GAAGAACCATTTGGAGATCTTGGAGAGGGCATTCTTTTCCTTGGGAATCTTAGAGGAAAGAGGGAGGATGTTTTTGCCAA

Yb1_cDNA_K326  561 ACTTCAGAGTCAGTTATCAGAAATTATGGGTGATAAGTACAACCTGTTCATGGTGGAGGAACCTAATTCAGAGGGGCCAG
Yb1_cDNA_TN90  561 ACTTCAGAGTCAGTTATCAGAAATTATGGGTGATAAGTACAACCTGTTCATGGTGGAGGAACCTAATTCAGAGGGGCCAG
Yb2_cDNA_K326  552 ACTTCAGAGTCAGTTATCAGAAATTATGGGTGATAAGTACAACCTGTTCATGGTGGAAGAGCCTAATTCAGAGGGGCCAG
Yb2_cDNA_TN90  552 ACTTCAGAGTCAGTTATCAGAAATTATGGGTGATAAGTACAACCTGTTCATGGTGGAAGAGCCTAACTCT--------GG

Yb1_cDNA_K326  641 ACCCGCGTGGTGGGCCCAGAGTCAGCTTTGGTATGCTGCGGAAAGAAGTTTCTGAACCAGGTCCAACAACTCTCTGGCAA
Yb1_cDNA_TN90  641 ACCCGCGTGGTGGGCCCAGAGTCAGCTTTGGTATGCTGCGGAAAGAAGTTTCTGAACCAGGTCCAACAACTCTCTGGCAA
Yb2_cDNA_K326  632 ACCCACGTGGTGGGCCCAGAGTTAGCTTTGGTATGCTGCGGAAAGAAGTTTCTGAACCAGGTCCAACAACTCTCTGGCAA
Yb2_cDNA_TN90  624 ACCCACGTGGTGGGCCCAGAGTTAGCTTTGGTATGCTGCGGAAAGAAGTTTCTGAACCAGGTCCAACAACTCTCTGGCAA

Yb1_cDNA_K326  721 TATGTAATTGCTTTTCTGTTGTTCCTTCTCACTATTGGTTCCTCTGTGGAGCTAGGAATTGCATCTCAGATAACTCGCCT
Yb1_cDNA_TN90  721 TATGTAATTGCTTTTCTGTTGTTCCTTCTCACTATTGGTTCCTCTGTGGAGCTAGGAATTGCATCTCAGATAACTCGCCT
Yb2_cDNA_K326  712 TATGTAATTGCTTTTCTGTTGTTCCTTCTCACAATTGGTTCCTCTGTGGAGCTAGGAATTGCATCTCAGATAACTCGCCT
Yb2_cDNA_TN90  704 TATGTAATTGCTTTTCTGTTGTTCCTTCTCACAATTGGTTCCTCTGTGGAGCTAGGAATTGCATCTCAGATAACTCGCCT

Yb1_cDNA_K326  801 TCCTCCTGAGGTAGTTAAGTACTTTACTGATCCAAATGCAATTGAACCACCAGATATGCAGCTTTTATTACCGTTTGTGG
Yb1_cDNA_TN90  801 TCCTCCTGAGGTAGTTAAGTACTTTACTGATCCAAATGCAATTGAACCACCAGATATGCAGCTTTTATTACCGTTTGTGG
Yb2_cDNA_K326  792 TCCTCCTGAGGTAGTTAAGTACTTTACGGATCCAAATGCAATTGAACCACCAGATATGCAGCTTTTACTACCGTTTGTGG
Yb2_cDNA_TN90  784 TCCTCCTGAGGTAGTTAAGTACTTTACGGATCCAAATGCAATTGAACCACCAGATATGCAGCTTTTACTACCGTTTGTGG

Yb1_cDNA_K326  881 ATTCTGCTTTACCGTTGGCATATGGTGTGCTGGGTGTGCAGTTATTTCATGAAATTGGGCATTTTCTGGCTGCATTTCCA
Yb1_cDNA_TN90  881 ATTCTGCTTTACCGTTGGCATATGGTGTGCTGGGTGTGCAGTTATTTCATGAAATTGGGCATTTTCTGGCTGCATTTCCA
Yb2_cDNA_K326  872 ATTCTGCTATACCACTGGCATATGGTGTGTTGGGCGTGCAGTTATTTCATGAAATTGGGCATTTTCTGGCTGCGTTTCCA
Yb2_cDNA_TN90  864 ATTCTGCTATACCACTGGCATATGGTGTGTTGGGCGTGCAGTTATTTCATGAAATTGGGCATTTTCTGGCTGCGTTTCCA

Yb1_cDNA_K326  961 AGGAATGTGAAATTAAGCATTCCTTTCTTTATTCCAAACATCACTCTTGGAAGCTTTGGAGCAATCACTCAGTTCAAATC
Yb1_cDNA_TN90  961 AGGAATGTGAAATTAAGCATTCCTTTCTTTATTCCAAACATCACTCTTGGAAGCTTTGGAGCAATCACTCAGTTCAAATC
Yb2_cDNA_K326  952 AGGAATGTGAAATTAAGCATTCCTTTCTTTATTCCAAACATCACTCTTGGAAGCTTTGGAGCAATCACTCAGTTCAAATC
Yb2_cDNA_TN90  944 AGGAATGTGAAATTAAGCATTCCTTTCTTTATTCCAAACATCACTCTTGGAAGCTTTGGAGCAATCACTCAGTTCAAATC

Yb1_cDNA_K326 1041 TATTCTTCCCGATCGCAAAGCAAAGGTAGACATTTCTCTTGCGGGTCCTTTTGCTGGTGCTGCATTGTCTTCTTCCATGT
Yb1_cDNA_TN90 1041 TATTCTTCCCGATCGCAAAGCAAAGGTAGACATTTCTCTTGCGGGTCCTTTTGCTGGTGCTGCATTGTCTTCTTCCATGT
Yb2_cDNA_K326 1032 TATTCTTCCTGATCGAAAAGCAAAGGTAGATATTTCGCTTGTGGGTCCTTTTGCTGGTGCTGCATTGTCTTCTTCAATGT
Yb2_cDNA_TN90 1024 TATTCTTCCTGATCGAAAAGCAAAGGTAGATATTTCGCTTGTGGGTCCTTTTGCTGGTGCTGCATTGTCTTCTTCAATGT
```

```
Yb1_cDNA_K326 1121 TTGCGGTTGGCCTGTTACTCTCATCCAATCCTGCTGCTGCTGGAGAGTTGGTTCAGGTTCCTAGCACACTTTTCCAGGGC
Yb1_cDNA_TN90 1121 TTGCGGTTGGCCTGTTACTCTCATCCAATCCTGCTGCTGCTGGAGAGTTGGTTCAGGTTCCTAGCACACTTTTCCAGGGC
Yb2_cDNA_K326 1112 TTGCGGTTGGCCTGTTACTCTCATCCAATCCTGCTGCTTCTGGAGAGTTGGTTCAGGTTCCTAGCACACTTTTCCAGGGA
Yb2_cDNA_TN90 1104 TTGCGGTTGGCCTGTTACTCTCATCCAATCCTGCTGCTTCTGGAGAGTTGGTTCAGGTTCCTAGCACACTTTTCCAGGGA

Yb1_cDNA_K326 1201 TCTTTGCTTCTCGGGCTTATTAGCAGAGCCACTCTTGGTTATGGAGCAATGCATGGTGCAATGGTTTCAATCCATCCTCT
Yb1_cDNA_TN90 1201 TCTTTGCTTCTCGGGCTTATTAGCAGAGCCACTCTTGGTTATGGAGCAATGCATGGTGCAATGGTTTCAATCCATCCTCT
Yb2_cDNA_K326 1192 TCTTTGCTTCTTGGGCTTATTAGCAGAGCCACTCTTGGTTATGGAGCAATGCATGGAGCAATGGTTTCAATCCATCCTCT
Yb2_cDNA_TN90 1184 TCTTTGCTTCTTGGGCTTATTAGCAGAGCCACTCTTGGTTATGGAGCAATGCATGGAGCAATGGTTTCAATCCATCCTCT

Yb1_cDNA_K326 1281 TGTGATAGCTGGCTGGTGTGGCTTGACTACATCGGCTTTTAATATGCTGCCAGTTGGATGTCTTGATGGTGGGAGAGCTG
Yb1_cDNA_TN90 1281 TGTGATAGCTGGCTGGTGTGGCTTGACTACATCGGCTTTTAATATGCTGCCAGTTGGATGTCTTGATGGTGGGAGAGCTG
Yb2_cDNA_K326 1272 TGTGATTGCTGGCTGGTGTGGTTTGACTACGTCGGCTTTTAATATGCTACCAGTTGGATGTCTTGATGGTGGGAGAGCTG
Yb2_cDNA_TN90 1264 TGTGATTGCTGGCTGGTGTGGTTTGACTACGTCGGCTTTTAATATGCTACCAGTTGGATGTCTTGATGGTGGGAGAGCTG

Yb1_cDNA_K326 1361 TGCAGGGAGCCTTTGGGAAAGGATCACTTATTGGTTTTGGTTTGGCGACATACACACTTCTGGGCTTGGGCGTGCTTGGT
Yb1_cDNA_TN90 1361 TGCAGGGAGCCTTTGGGAAAGGATCACTTATTGGTTTTGGTTTGGCGACATACACACTTCTGGGCTTGGGCGTGCTTGGT
Yb2_cDNA_K326 1352 TGCAGGGAGCCTTTGGGAAAGGATCACTTATTGGTTTTGGTTTGGCGACATACACACTTCTGGGCTTGGGCGTGCTTGGT
Yb2_cDNA_TN90 1344 TGCAGGGAGCCTTTGGGAAAGGATCACTTATTGGTTTTGGTTTGGCGACATACACACTTCTGGGCTTGGGCGTGCTTGGT

Yb1_cDNA_K326 1441 GGACCTC-TGTCACTTCCTTGGGGATTGTATGTGCTTATATGTCAGAGGACACCGGAGAAACCATGCTTGAATGATGTAA
Yb1_cDNA_TN90 1441 GGACCTCTTGTCACTTCCTTGGGGATTGTATGTGCTTATATGTCAGAGGACACCGGAGAAACCATGCTTGAATGATGTAA
Yb2_cDNA_K326 1432 GGACCTC-TGTCACTTCCTTGGGGATTATATGTGCTTATATGTCAGAGGACACCAGAGAAACCATGCTTGAACGATGTAA
Yb2_cDNA_TN90 1424 GGACCTC-TGTCACTTCCTTGGGGATTATATGTGCTTATATGTCAGAGGACACCAGAGAAACCATGCTTGAACGATGTAA

Yb1_cDNA_K326 1520 CAGAGGTCGGAAATTGGAGAAAAGCAGCTCTTGGTGTGGCTATATTCCTTGTTGTATTGACTCTTCTTCCTGTATGGGAT
Yb1_cDNA_TN90 1521 CAGAGGTCGGAAATTGGAGAAAAGCAGCTCTTGGTGTGGCTATATTCCTTGTTGTATTGACTCTTCTTCCTGTATGGGAT
Yb2_cDNA_K326 1511 CAGAGGTCGGAACTTGGAGAAAAGCAGCTCTTGGTGTGGCTATATTCCTTGTAGTATTGACTCTTCTTCCTGTATGGGAT
Yb2_cDNA_TN90 1503 CAGAGGTCGGAACTTGGAGAAAAGCAGCTCTTGGTGTGGCTATATTCCTTGTAGTATTGACTCTTCTTCCTGTATGGGAT

Yb1_cDNA_K326 1600 GAACTTGCAGAAGAACTAGGTATAGGTCTTGTAACCAGCTTTTGA
Yb1_cDNA_TN90 1601 GAACTTGCAGAAGAACTAGGTATAGGTCTTGTAACCAGCTTTTGA
Yb2_cDNA_K326 1591 GAACTTGCAGAAGAACTAGGTATAGGTCTTGTAACCAGCTTTTGA
Yb2_cDNA_TN90 1583 GAACTTGCAGAAGAACTAGGTATAGGTCTTGTAACCAGCTTTTGA
```

Supplemental Data 6. cDNA alignments of *Yb* genes. Alingments were generated using Vector-NTI AlignX. Conserved nucleotides are indicated by shaded squares. The mutations in TN 90 which result in the yellow burley phenotype are underlined with solid gray lines.
