# Supplementary material for: A reference genome for Nicotiana tabacum enables map-based cloning of homeologous loci implicated in nitrogen utilization efficiency
Source: BMC Genomics. 2017 Jun 19;18:448. doi: 10.1186/s12864-017-3791-6 (PMC5474855; doi:10.1186/s12864-017-3791-6)
Supplement: Supplementary file 7 — Phylogenetic analysis of predicted protein sequences for EGY1 homologs in various plant species. (PDF 303 kb) [file 12864_2017_3791_MOESM7_ESM.pdf]

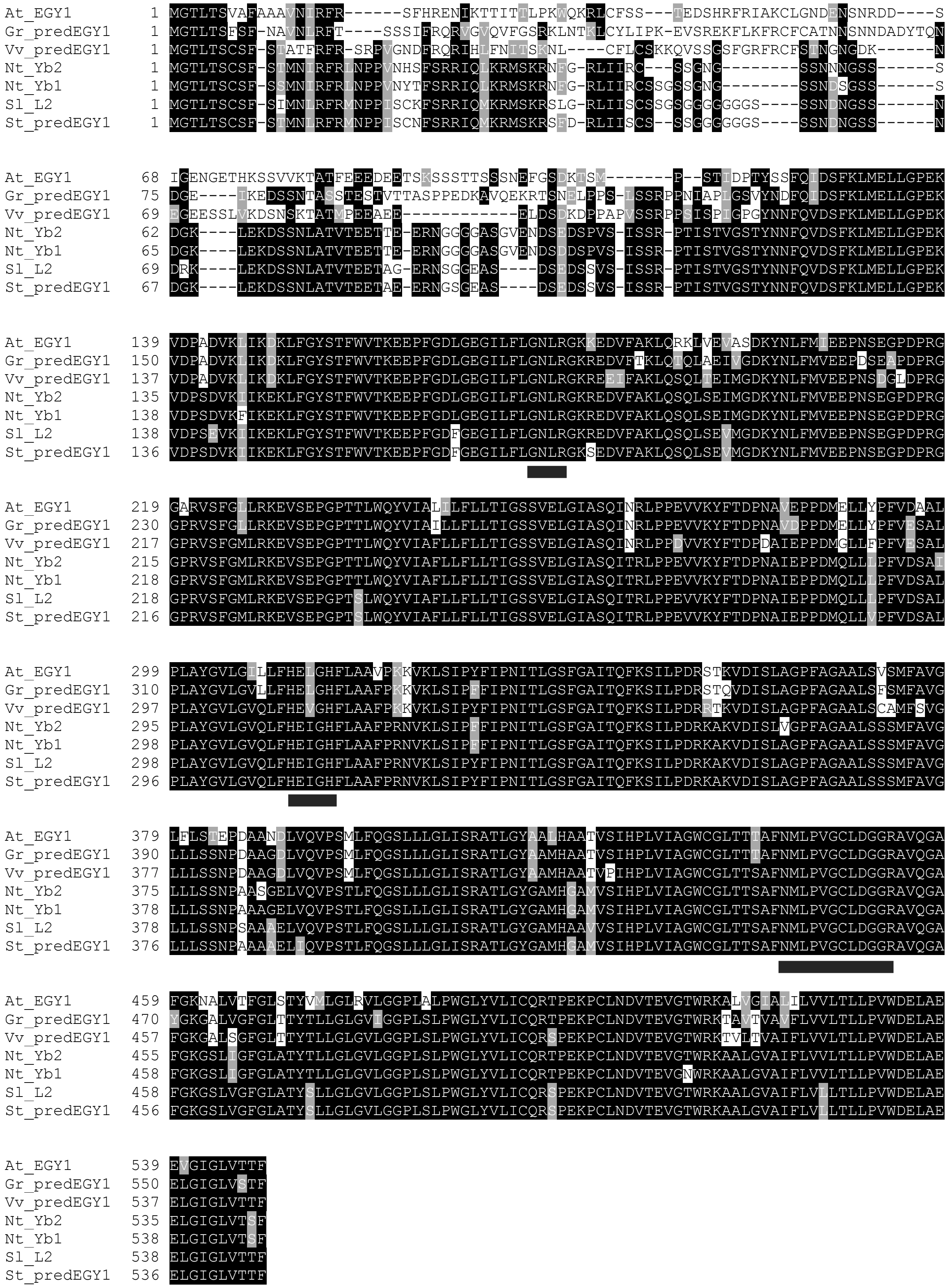

Amino acid alignments of Yb related proteins. Alignments were generated using MUSCLE. Conserved nucleotides are indicated by shaded squares. The three conserved domains, GNLR, HEXXH, and NPDG are underlined with black lines. Accession numbers are as follows: *Arabidopsis* EGY1 (NP_198372), *Gossypium raimondii* predicted EGY1 (XP_012492480), *Vitis vinifera* predicted EGY1 (XP_002269447), *S. lycopersicum* L2 (NP_001299816), *S. tuberosum* predicted EGY1 (XP_006339202).

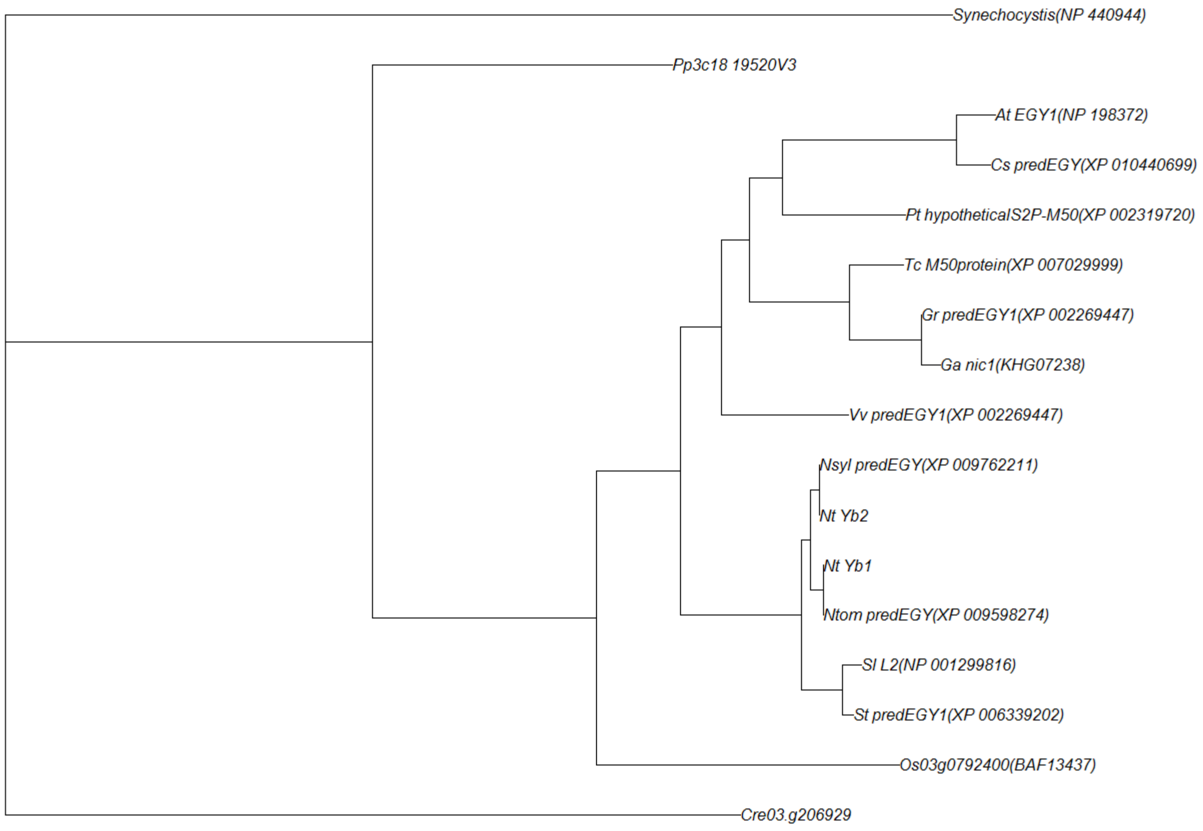

Phylogeny of Yb_1 and Yb_2 related proteins. The dendrogram was constructed using the maximum likelihood method. GenBank accession number are given for each branch except for the Cre03.g206929 and Pp3c18_19520V3, which are based on annotations of the *Chlamydomonas reinhardtii* and *Physcomitrella patens* genomes available through the Phytozome database (www.phytozome.net).
