# Supplementary material for: A reference genome for Nicotiana tabacum enables map-based cloning of homeologous loci implicated in nitrogen utilization efficiency
Source: BMC Genomics. 2017 Jun 19;18:448. doi: 10.1186/s12864-017-3791-6 (PMC5474855; doi:10.1186/s12864-017-3791-6)
Supplement: Supplementary file 8 — Expression pattern of EGY1 genes in tobacco and Arabidopsis. (PDF 234 kb) [file 12864_2017_3791_MOESM8_ESM.pdf]

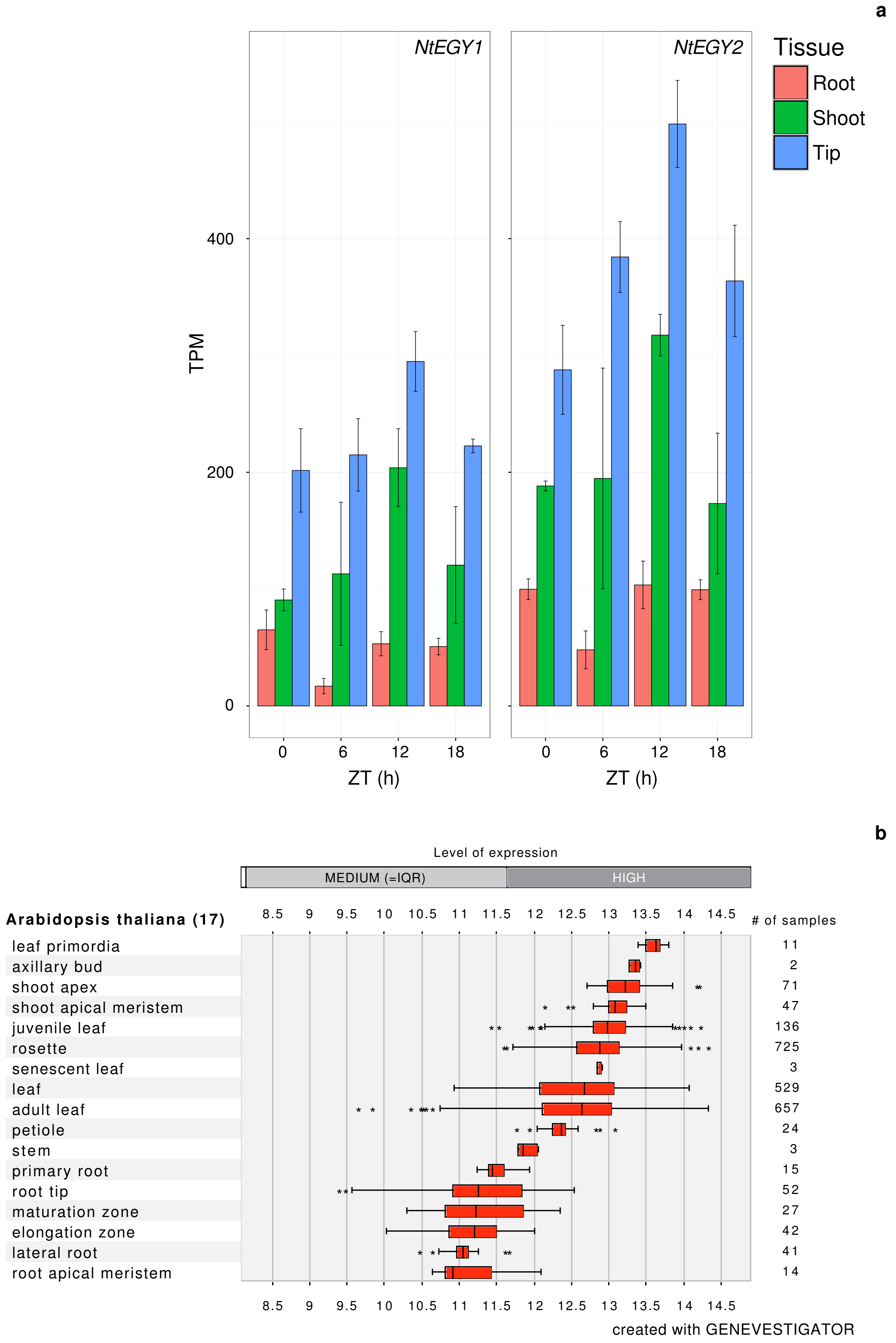

Supplementary Data 9 - *EGY1* gene expression in tobacco and Arabidopsis

a, Expression (TPM) of *NtEGY1* and *NtEGY2* genes in *N. tabacum* (cv. K326) from root (red), shoot (green) and shoot-tip (blue) samples taken at 0, 6, 12, and 18 hours after dawn (ZT).

b, Expression of *AtEGY1* across multiple tissues based on Affymetrix ATH1 microarray data exported from Genevestigator showing higher expression in shoot apices and leaf samples versus root samples.
