# Supplementary material for: A reference genome for Nicotiana tabacum enables map-based cloning of homeologous loci implicated in nitrogen utilization efficiency
Source: BMC Genomics. 2017 Jun 19;18:448. doi: 10.1186/s12864-017-3791-6 (PMC5474855; doi:10.1186/s12864-017-3791-6)
Supplement: Supplementary file 9 — Table showing genotypic analysis of tobacco cultivars. (PDF 22 kb) [file 12864_2017_3791_MOESM9_ESM.pdf]

Supplementary Data 8. Genotypic survey of tobacco cultivars from different market classes.
for the *Yb* loci.

Line	Market class[1]	Accession Number[2]	TC Number[3]	Stem Color	*YB1* Genotype[4]	*YB2* Genotype[5]
Posey	Maryland	PI 552743	512	Green	*YB1*	*YB2*
Catterton	Maryland	PI 551332	494	Green	*YB1*	*YB2*
Green Brior	Burley	PI 552645	40	Green	*YB1*	*YB2*
Gertz	Maryland	PI 552641	496	Green	*YB1*	*YB2*
Sweeney	Maryland	PI 552762	514	Green	*YB1*	*YB2*
Moore	Maryland	PI 552703	511	Green	*YB1*	*YB2*
Wilson	Maryland	PI 551341	517	Green	*YB1*	*yb2*
Md 10	Maryland	PI 551333	498	Green	*YB1*	*yb2*
Md 59	Maryland	PI 552451	501	Green	*YB1*	*yb2*
Md 64	Maryland	PI 551334	502	Green	*YB1*	*yb2*
Md 609	Maryland	PI 552452	505	Green	*YB1*	*yb2*
Barnett Special	Burley	PI 552589	1	White	*yb1*	*yb2*
Judy's Pride	Burley	PI 552483	49	White	*yb1*	*yb2*
Kelly Brownleaf	Burley	PI 552670	50	White	*yb1*	*yb2*
Kelly Burley	Burley	PI 552671	51	White	*yb1*	*yb2*
Burley 1	Burley	PI 552360	3	White	*yb1*	*yb2*
Burley 2	Burley	PI 552361	4	White	*yb1*	*yb2*
Burley 11A	Burley	PI 552362	5	White	*yb1*	*yb2*
Burley 21	Burley	PI 552363	7	White	*yb1*	*yb2*
Ky 1	Burley	PI 552673	52	White	*yb1*	*yb2*
Ky 5	Burley	PI 551278	53	White	*yb1*	*yb2*
Ky 9	Burley	PI 552365	54	White	*yb1*	*yb2*
Ky 14	Burley	PI 552477	57	White	*yb1*	*yb2*
Ky 16	Burley	PI 552366	59	White	*yb1*	*yb2*

[1] According to USDA GRIN database

[2] USDA GRIN database

[3] 'TC' indicates 'Tobacco Cultivar in the United States *Nicotiana* Germplasm Collection.

[4] Determined by testing for for 1 bp insertion discovered in burley tobacco cultivar TN 90.

[5] Determined by testing for for 8 bp deletion mutation discovered in burley tobacco culitvar TN 90.
