# Supplementary material for: A reference genome for Nicotiana tabacum enables map-based cloning of homeologous loci implicated in nitrogen utilization efficiency
Source: BMC Genomics. 2017 Jun 19;18:448. doi: 10.1186/s12864-017-3791-6 (PMC5474855; doi:10.1186/s12864-017-3791-6)
Supplement: Supplementary file 10 — Table summarising sequence data used in tobacco genome assembly. (PDF 289 kb) [file 12864_2017_3791_MOESM10_ESM.pdf]

Supplementary Data 10 – Tobacco Genome sequence data

NGS Assembly

Sequence Class	No. reads	No. bases	Depth*	Genbank accession numbers (SRA)
454 SG	64,356,541	23,232,283,876	5.16	SRR5275650 – SRR5275754
454 SG XL	52,901,706	32,780,428,773	7.28	SRR5275610 – SRR5275649
454 PE3	35,783,818	10,877,165,346	2.42	SRR5275795 – SRR5275866
454 PE8	20,303,227	5,910,090,732	1.31	SRR5275755 – SRR5275794
454 PE20	18,783,288	5,766,515,000	1.28	SRR5275867 – SRR5275892
**454 Total**	**192,128,580**	**78,566,483,727**	**17.46**	
PE 300bp	1,091,120,572	109,112,057,200	24.25	SRR5277616, SRR5277617, SRR5277619, SRR5277622, SRR5277624, SRR5277625, SRR5277628, SRR5277629, SRR5277631
MP 2kbp	160,849,530	16,245,802,530	3.61	SRR5277613
MP 8kbp	1,008,919,040	98,874,065,920	21.97	SRR5277607, SRR5277609, SRR5277611
**Illumina Total**	**2,471,801,010**	**308,564,924,377**	**68.57**	
**Grand Total**		**387,131,408,104**	**86.03**	

Table showing the number of reads and total bases of each class that were included in the tobacco genome assembly. Note that the Roche 454 data represents raw sequence reads and the Illumina data represents filtered sequence reads (See methods).
* Depth was calculated based on an estimated genome size of 4.5Gbp.
