# Supplementary material for: A reference genome for Nicotiana tabacum enables map-based cloning of homeologous loci implicated in nitrogen utilization efficiency
Source: BMC Genomics. 2017 Jun 19;18:448. doi: 10.1186/s12864-017-3791-6 (PMC5474855; doi:10.1186/s12864-017-3791-6)
Supplement: Supplementary file 11 — Tables summarising primer sequences used in cloning and genotyping analysis. (PDF 282 kb) [file 12864_2017_3791_MOESM11_ESM.pdf]

Table A – KASP design regions for *yb1 yb2* fine mapping

Marker name	DNA sequence
Yb5-1	TTAATAATAGTTCCTTTTTGACCGCGGGTACGAGGGCTAGCGTGAAATTGGCCACGAAGT[T/C]TGCCTAGATTTGAGACTTAATAGACGTTCGGGGTTGATACTCAATGTCGTACCTACTAAT
Yb5-4	GACTACTATCATTGCCAGTCTCAACCTGTTCTGGTTCAAGCTTCAGTTCCACGTCCTCTC[T/C]ATCAGTCTCTGCAGGCTCAACTGTTTCGATCTCCTTGTGTTCTGGAACTTCTTTAGAATC
Yb24-1	TAGATATTTGCCTCTTGTAGCTGGTTTACCATTTATAAAGCTTTTTTCCTGTTGCCGCAT[A/G]ATGAAGTGTAATGTTGTCTCTATTATCTGTTAAAGACTTGTGATGTAGCTTGGTGTTCTG
Yb24-2	GAATCCTATGCTGGGATTAAGCCAAGCTGGTTTAGCTGCATCGGGATTTAATCCTTCGTT[T/C]GTGGGGATTGGTGCTGGTTATGATATAAATAGCATTAATCCGGGCGTTTTAGGTAGTAGT
Yb24-4	GTGGAATCTAAGGAGGGTAAAGTGTAAGCAAGTTTATCCTTGTCCTAGAAAGGTAGAGAT[A/G]TTGTTTCCGATAGATCCTTGGCTAAAAAAACGCAGAAAAGAAGCAGTGTCAACAAGTAAT
Yb24-6	GCAATATAAAAGTCAATACTAGTCTTAACGGTGTGAGGCCTTTTGGAAAAACTTGTACGG[T/G]CTTGGTCCAAAACAGACAATATCACACCATTTAAGAGTATCTTTAGGCCGTTTTAGTCCG
Yb24-7	GACGGTATGGTTAAAGATGTATATACGGAAGACTTCTCGATCACCATCGGAGAGAAATGC[T/G]TATTTTTATGTCTCTTTTGGCCCAATGGAGAGCCCTTATTTATAAATGTAGAAGATTGAA
EGY_2kasp	GCTTAGTGTTGCAAATTGTACAGTCACTTAAAACATTCTGTTTGTGCTGCTATCAGCTTGGTGGACCTCT[T/]GTCACTTCCTTGGGGATTGTATGTGCTTATATGTCAGGTAATAAAAATACATTGCTTTATGAAGTATTATGCTTGTGTAATAATATCGCCGTCCTATTTCATGGTATA

Table B – Oligonucelotides used for map-based identification of yb1 yb2 genomic DNA and cDNA

Task	Oligonucleotides Used
Genotyping of EGY1 deletion	EGY_1-F/EGY_1-R
Genotyping of EGY1 homeolog insertion	EGY_2-nF/EGY_2-nR followed by EGY_2-kasp
Amplification of Yb1 cDNA	cYb-F/cYb-R
Amplification of Yb2 cDNA	cYb-F/cYb-R
Amplification of Yb2 genomic fragment 1	gYb2-F1/gYb2-R1
Internal sequencing of Yb2 genomic fragment 1	gYb2_Int1
Amplification of Yb2 genomic fragment 2	gYb2-F2/gYb2-R2
Amplification of Yb2 genomic fragment 3	gYb2-F3/gYb2-R3
Internal sequencing of Yb2 genomic fragment 3	gYb2-Int3
Amplification of Yb2 genomic fragment 4	gYb2-F4/gYb2-R4
Amplification of Yb2 genomic fragment 5	gYb2-F5/gYb2-R5
Amplification of Yb2 genomic fragment 6	gYb2-F6/gYb2-R6
Amplification of Yb1 genomic fragment 1	gYb1-F1/gYb1-R1
Internal sequencing of Yb1 genomic fragment 1	gYb1-Int1
Amplification of Yb1 genomic fragment 2	gYb1-F2/gYb1-R2
Amplification of Yb1 genomic fragment 3	gYb1-F3/gYb1-R3
Amplification of Yb1 genomic fragment 4	gYb1-F4/gYb1-R4
Amplification of Yb1 genomic fragment 5	gYb1-F5/gYb1-R5
Internal sequencing of Yb1 genomic fragment 5	gYb1-Int5
Amplification of Yb1 genomic fragment 6	gYb1-F6/gYb1-R6
Internal sequencing of Yb1 genomic fragment 6	gYb1-Int6
Amplification of CRISPR/Cas Yb2 target site 1	gYb2-F1/gYb2-R1
Sequencing of CRISPR/Cas Yb2 target site 1	gYb2-F1
Amplification of CRISPR/Cas Yb2 target site 2	gYb2-F1/gYb2-R2
Sequencing of CRISPR/Cas Yb2 target site 2	gYb2-CC2
Amplification of CRISPR/Cas Yb1 target site 1	gYb1-F1/gYb1-R2
Sequencing of CRISPR/Cas Yb1 target site 1	gYb1-Int1
Amplification of CRISPR/Cas Yb1 target site 2	gYb1-F1/gYb1-R1
Sequencing of CRISPR/Cas Yb1 target site 2	gYb1-F1
Amplification of fragment with Yb2 deletion in Maryland lines	gYb2-F2/gYb2-R2
Sequencing of Yb2 deletion fragment in Maryland lines	gYb2-F2
Amplification of fragment with Yb1 insertion in Maryland lines	gYb1-F6/gYb1-R6
Sequencing of Yb1 insertion fragment in Maryland lines	gYb1-6

Table C – Oligonucleotide sequences used in map-based cloning of yb1 and yb2 genomic DNA and cDNA

Primer Name	Primer Sequence (in 5' to 3' direction)
EGY1-F	AAGAGTAGCTGCATGATGTGAAGA
EGY1-R	CTGGCCCCTCTGAATTAGGTTCCTC
EGY2-nF	GCAATCGTTGTCCAGTGTCTA
EGY2-nR	TTTCCGACCTCTGTTACATCA
cYb-F	ATGGGAACGCTAACGAGCTGCAGTTTCAGCACAATGAAT
cYb-R	TCAAAAGCTGGTTACAAGACCTATACCTAGTTCTTCTGCAAG
gYb2-F1	ATGGGAACGCTAACGAGCTGCAGTTTCAGCA
gYb2-R1	CCGTGGATATTGTTGGCTGCCAAGAAATT
gYb2-Int1	CCGGTGTCAATTTCTTCCAGA
gYb2-F2	AAGGTTGATCCCAGTGATGTGAAGATA
gYb2-R2	CCAACACACCATATGCCAGTGGTAT
gYb2-F3	CCGTTTGTGGATTCTGCTATACCAC
gYb2-R3	GGAATGCTTAATTTCACATTCCTTGGAAAC
gYb2-Int3	CTGATTTAGCTATGCTAGTTTGC
gYb2-F4	GAAATTGGGCATTTTCTGGCTGCG
gYb2-R4	GCAAAAGGACCCACAAGCGAAATA
gYb2-F5	CGAAAAGCAAAGGTAGATATTTCGCTTGT
gYb2-R5	CCAATAAGTGATCCTTTCCCAAAGGC
gYb2-F6	GCCTTTGGGAAAGGATCACTTATTGG
gYb2-R6	CCACTACGTACAGTCAAAAGATCAGTATGAA
gYb1-F1	CCTCACCATCTTCCAATTACATTTTTCACCATTG
gYb1-R1	CACATTCAGTACATTGGTTTCTCATCTTCATCTG
gYb1-Int1	AAGAAGCTCGTCACATGCATTC
gYb1-F2	AAGGTTGATCCCAGTGATGTGAAGTTC
gYb1-R2	CCAACACACCATATGCCAACGGTAA
gYb1-F3	CCGTTTGTGGATTCTGCTTTACCGT
gYb1-R3	GGAATGCTTAATTTCACATTCCTTGGAAAT
gYb1-F4	GAAATTGGGCATTTTCTGGCTGCA
gYb1-R4	GCAAAAGGACCCGCAAGAGAAATG
gYb1-F5	CGAAAAGCAAAGGTAGACATTTCTCTTGC
gYb1-R5	GGTGTCCAATTTCCCTAGCCG
gYb1-Int5	GGTTTCAATCCATCCTCTTGTGAT
gYb1-F6	CGGCTAGGGAAATTGGACACC
gYb1-R6	CCACTACGTACAGTCAAAAGATCAGTATGAT
gYb1-Int6	CTCCGCCATCAAACACCGA
gYb2-CC2	GGTTTAAAAGAATTGTGGCTGGGGTA
